# Local traditional ecological knowledge about hay management practices in wetlands of the Biebrza Valley, Poland

**DOI:** 10.1186/s13002-022-00509-9

**Published:** 2022-02-22

**Authors:** Joanna Sucholas, Zsolt Molnár, Łukasz Łuczaj, Peter Poschlod

**Affiliations:** 1 Ecology and Conservation Biology, Institute of Plant Sciences, University of Regensburg, Universitätsstr. 31, 93053 Regensburg, Germany; 2 University of Applied Forest Sciences, Schadenweilerhof, 72108 Rottenburg am Neckar, Germany; 3 Centre for Ecological Research, Institute of Ecology and Botany, Vácrátót, 2163 Hungary; 4 Institute of Biology and Biotechnology, University of Rzeszów, ul. Pigonia 1, 35-310 Rzeszów, Poland

**Keywords:** Ethnoecology, Traditional management, Knowledge preservation, Cultural landscape, Conservation area, Lowland river meadows

## Abstract

**Background:**

The Biebrza Valley is one of the largest complexes of wetlands (floodplain and percolation mire) and conservation sites in Central Europe. Local communities have managed the area extensively for subsistence and farming purposes for centuries; nonetheless, since the 1960s, hand mowing and livestock grazing have been gradually ceasing due to the intensification of farming, and wetlands have undergone natural succession. Currently, the protection of this vast ecosystem is challenging. Despite its remarkable cultural origin, the complexity of the traditional practices and knowledge of local people have never been studied comprehensively. Therefore, we found it urgent to explore if traditional ecological knowledge that could be used in conservation management of the area still exists among the local community.

**Methods:**

We interviewed 42 inhabitants of seven villages located in the Lower Basin of the Biebrza Valley (NE-Poland) in the consecutive years 2018–2020. We applied semi-structured, repeated interviews with farmers (aged 29–89), each lasting several hours. By using different ethnoecological methods (visual stimuli, walks in wetlands, co-mapping of the area), we explored traditional knowledge on the plants, landscape and traditional management of wetlands.

**Results:**

Farmers from the oldest generation, who used to manage wetlands with scythes, shared the deepest ecological knowledge. Local people divided wetlands into zones differentiated by vegetation type and hay quality. Depending on plant composition, people managed wetlands under a mixed regime: mowing once or twice a year during periods that ensured good hay quality and pasturing various livestock: cattle, horses, sheep, pigs and fowl. We identified at least 50 plant ethnospecies, which were described exhaustively by their habitat, morphological features, and mowing and grazing value.

**Conclusions:**

The local community in the Biebrza Valley shared a deep traditional ecological knowledge and had a good memory of traditional farming practices. Research confirmed the unquestionable cultural origin of the local ecosystem, therefore in conservation endeavours the area should be treated first and foremost as a cultural landscape. The documented exceptional local perception of the wetland landscape, elements of traditional knowledge and complex farming practices should be considered for inclusion into conservation management, and cooperation with the local community should also be taken into account.

## Introduction

In the last decades, many studies have revealed the presence of deep knowledge of natural habitats and their management among rural communities in Europe [1–6]. Traditional ecological knowledge (TEK) was previously widely recognised by scientists mainly among indigenous tribal communities outside Europe [7–10] and others. Studies on TEK provide insights into the relationship of local human communities with surrounding nature and into people’s understanding of the interactions between elements of nature. TEK evolves throughout the centuries-long life experience of a community within a certain environment and is orally transmitted from generation to generation. TEK incorporates knowledge about elements of the environment, beliefs, ethical values and human practices [11–13] which are the objects of ethnoecological and allied studies [14–16]. However, this knowledge differs in the context of living European rural communities, which were not colonised, derive from the same Judeo-Christian tradition and whose culture was impacted by Roman and Greek heritage. Additionally, it can be influenced by globalisation, agribusiness [17] and education. Molnár [18] defines such traditional ecological knowledge as locally embedded, empirical, ‘*based upon decades of personal experience with the surrounding landscape, acquired through hands-on management of the landscape, containing centuries-old, communally stored experiences which is mostly independent of western science and connected to rituals of social life*’. Such knowledge is locally relevant and applicable, therefore we will name it local traditional ecological knowledge (LTEK).

Research shows that European local communities—either farmers, herders, foresters or fishermen by their traditional practices—have co-created biodiverse habitats, such as high-value grasslands [19–21], and often continue to maintain them [4, 17, 22]. Similarly, the application of traditional practices has led to the development of some wetland types in Europe [23–30]. European wetlands have been traditionally used for grazing, haymaking, hunting, burning, fishing, reed cutting, etc. [31–34]. Owing to that, researchers call for studies in Europe to explore local TEK about landscape, natural resources, land-use practices and their changes, which, when integrated, could help in local conservation and land management endeavours [35]. Moreover, the benefits of such studies for nature conservation are already widely discussed and acknowledged on a global [36–43] and European scale [3, 6, 30, 44–48].

Despite the significant share of human-made and human-managed habitats in Europe [45, 49, 50], cultural landscapes are vanishing, and traditional knowledge and practices are often abandoned [51–56] due to socio-economical and technical changes of land use, farming intensification, the lack of a traditional management-inclusive policy or the establishment of protected areas that restrict the application of traditional practices. The discontinuation of traditional practices endangers high value habitats and their biodiversity [46, 57]. In the case of wetlands, the common drainage practice for agricultural intensification is the major threat [24, 58–60] along with natural succession caused by land use abandonment [32, 61]. First, these processes degrade wetland vegetation and their biodiversity [58, 62, 63]. Second, drainage is the main reason for the eradication of related traditional knowledge, which is no longer implemented [64, 65].

However, even if the traditional farming in Europe is not sustained, it should be feasible to reconstruct it (and the associated knowledge) through the analysis of ecological, archaeological, ethnographic and historical materials [33, 34, 51, 66] or by interviewing local communities, especially the oldest generation, who could still store such knowledge in their memory [35]. The studies show that knowledge might differ according to generation, gender and other variables [67]. In Europe, there are cases of successful nature conservation or restoration by the implementation of traditional practices, for example, the traditional management of subalpine meadows ‘Mähder’ in Switzerland, supported by a subsidy system [68]; the conservation of meadow orchards ‘Strauobstwiesen’ in Germany, regulated by local policy [69]; restoration projects of German wetlands [24, 70]; traditionally used floodplains of the Sava River in Lonjsko Polje Nature Park in Croatia [26].

The Biebrza Valley in NE Poland (RAMSAR and NATURA 2000 Site) is one of the largest high value wetlands of cultural origin in Central Europe. The valley is the biggest conservation area of alkaline fens in the EU that needs to be managed to prevent overgrowing [71]. Over centuries, swamps, fens and flooded marshes in the Biebrza Valley were used by peasants for haymaking and grazing. Starting from the 1960s, a part of the wetlands around the Biebrza Valley was drained to facilitate the intensification of agriculture, which caused the retreat of some farmers in the undrained areas [72, 73]. The abandonment of wetland use led to shrub and reed encroachment, endangering the biodiverse open wetland habitats [62, 74]. Since the Biebrza National Park was established in 1993, conservationists have been undertaking activities to prevent the succession of vegetation (e.g. shrub removal, mowing with tracked mowers [71, 75]). However, restrictions have been introduced and farming, financially supported by Common Agriculture Policy, has been intensified after the accession of Poland to the EU in 2004. The general frame of wetland management is often defined by EU agri-environmental schemes [76]. All this could eradicate traditional knowledge of local people. Surprisingly, ethnographic studies from the area are scarce [77, 78] and only Kiryło [78] described, to some extent, the traditional practices on the wetlands and the locals’ knowledge in her ethnobiological Masters thesis. Thus, we recognise an urgent necessity for ethnoecological studies in this area. The study aims to identify what traditional knowledge about wetlands is possessed by the local community living in the Biebrza Valley and to discuss if this knowledge should be used in the management of the nature protection area. For these purposes, the following research questions have been formed:

1. Is LTEK still present among the local community and how is it distributed?
2. How do people perceive and value the wetland landscape?
3. How did they traditionally manage wetlands?
4. What knowledge do people have about plants occurring in the wetlands?

## Methods

### Research area

The Biebrza National Park located in Podlaskie Province, in NE Poland (53°28′00″N, 22°39′41″E), with a coverage of ca. 600 km^2^, protects wetland ecosystems in the Biebrza Valley [71]. The climate in the area is temperate continental, the average annual air temperature is 6.8 °C and the mean annual precipitation is 583 mm. The area has ca. 200 days of vegetation season [79]. The Biebrza Valley is a large floodplain depression with three distinguishable basins, covered mainly by wetlands (60%) with sandy ‘islands’ (40%). The valley is supplied by water from regular annual floods of the Biebrza River (extending up to even a few kilometres in width within the Lower Biebrza Basin [LBB]) and by groundwater running out from a morainic plateau on the other side of the valley in the direction of the Biebrza River. These hydrogeological conditions caused the development of organic-mineral and muddy soils along the river and peatlands in permanently watered areas [80]. Peatland types are typical floodplain and percolation mires [81].

In historical times, the area was covered by forests [82] with oak, lime and hornbeam on mineral islands and alder, birch and ash trees in swamps. Floodplain mires and marshes by the rivers were areas without trees [83]. Until the fourteenth century, the Biebrza Valley was used extensively and seasonally by hunters, fishermen, cattle herders, haymakers and beekeepers [84]. Permanent settlement started at the end of the fourteenth century, and villages developed on the elevated outskirts of the Biebrza Valley. For centuries, serf peasants used wetlands for haymaking and pasture farming, the river for fishing and the woodlands as a source of timber [85]; however, details concerning land management remain vague due to scarce ethnographic studies in the Biebrza Valley. The serf system was abolished progressively in the nineteenth century [86]. After the abolition of feudalism, the local community managed wetlands under various proprietary conditions, depending on the village and political system.

Since the times of the first settlement, Poles, *Rusini* (Orthodox Eastern Slavic dialect speakers) and Lithuanians inhabited this area [84, 85]. Over centuries, the Biebrza River in the LBB was a frontier between changeably governed countries (like e.g. the Polish Crown and Lithuania in 1385–1568 or the Congress of Poland and the Russian Empire in 1815–1917) or Polish Provinces (like Masovia and Podlasie in 1569–1795) [87]. Even though the whole Biebrza Valley has belonged to the Republic of Poland since 1918 and exclusively Catholic Poles live on both sides of the river in the LBB [88], remnants of the historical borders are still present in the self-perception of the local community. All informants living on the right side of the Biebrza River state: *behind the river, there are Ruscy/Rusini, here, we are Mazury* (related to ethnic group of Poles—Masurians, the former name of Masovians). Informants also often called people living on the left side of the river *Podlachy* (related to the province *Podlasie*) or *Litwasy* (related to times of Lithuanian influence). All informants from the left side of Biebrza confirm such naming. The same type of self-perception was one of the findings of Kiryło [78].

**Fig. 1:**
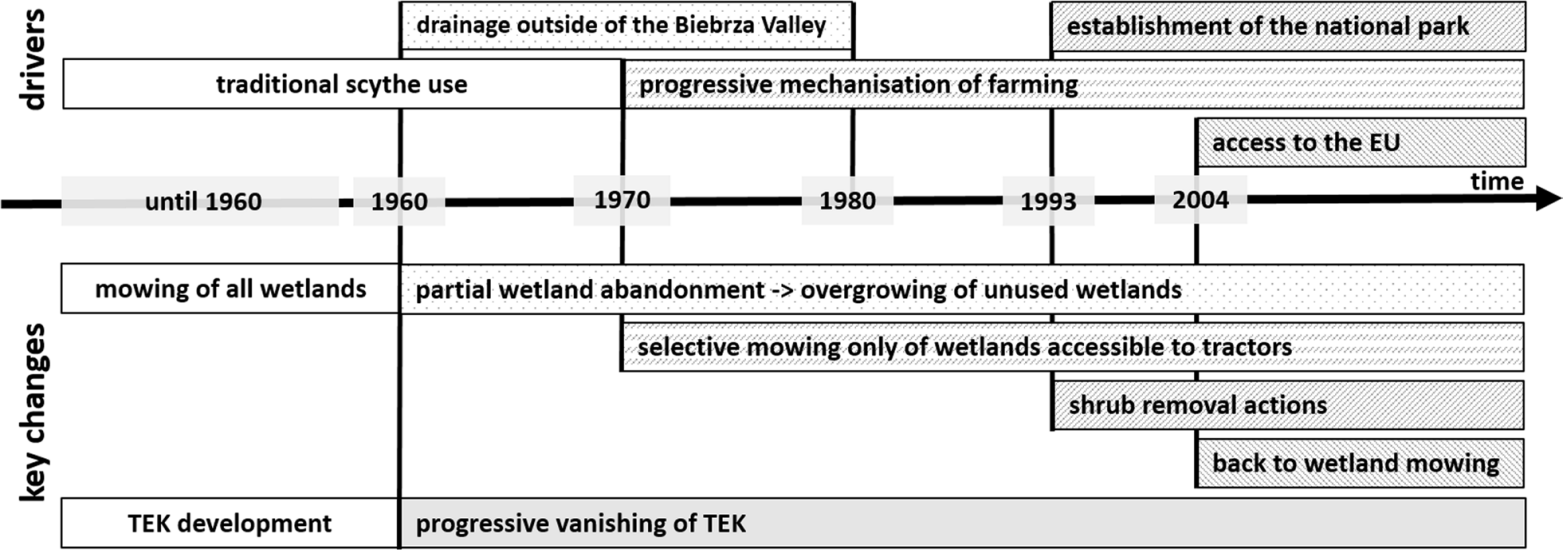
A timeline showing the key changes in the management and vegetation of wetlands and the main drivers behind these changes (Lower Biebrza Basin, Poland)

Owing to the fact that Biebrza wetlands functioned as a natural border, drainage was forbidden over the centuries. Large networks of channels (such as the Augustowski Canal) were built up in the second half of nineteenth century [87]. However, the floodplain of the LBB with its natural hydrological conditions remains well preserved to this day [89] and became the ultimate research area of this study. The wetlands of the LBB were collectively used not only by the local community but also by people living in distant villages (20–30 km away). However, people from distant villages have been progressively abandoning undrained wetlands of the LBB since the 1960s (Fig. 1). This was firstly due to the common drainage of land for agricultural purposes in the outskirts of the Biebrza Valley (between ca. 1960–1980), which resulted in the retreat of farmers from the undrained wetlands [90]. Later, along with process of farming mechanisation starting at the end of the 1960s, the use of the scythe was gradually discontinued [73]. The undrained wetlands suitable for scythe mowing were inaccessible for tractors [91]. Even though local inhabitants continued to use wetlands to some extent, a massive process of overgrowing with reed and willows has started in the LBB [71] and traditional practices have gradually ceased. Currently, the economy in the Biebrza Valley and the whole Podlaskie Province relies on agriculture and tourism; however, it is an economically marginalised region compared to other parts of Poland [92], and it is marked by mass emigration [93].

The vegetation in the LBB (an area with a length of 30 km and a width of 12–15 km [91]) has a zonal pattern that is formed parallel to the course of the river. The five vegetation zones are distinguished according to plant composition, source of water and duration of floods. Reedbeds and marshes have developed in the first zone, which is adjacent to the river and periodically intensively flooded. They are dominated by *Oenantho-Rorippetum, Glycerietum maximae* and *Caricetum gracilis* associations. The second zone consists of tall sedges, which form tussocks dominated by such communities as *Caricetum elatae, Caricetum rostratae* and *Caricetum appropinquatae.* The first and second zones are ca. 2 km-wide together. In the third zone, which is constantly supplied by groundwater, we find sedge-moss communities with species like *Carex appropinquata, C. nigra, C. panicea* (for vascular plant species author names see Table 5) and *Acrocladium cuspidatum* (Hedw.) Lindb. moss species. The fourth zone is never flooded, but it is watered by groundwater. Low-sedge-brown moss communities grow in this zone, with species like *Carex diandra, C. lasiocarpa, C. flava* and *Drepanocladus revolvens* (Sw.) Warnst., *Hamatocaulis vernicosus* (Mitt.) Hedenäs mosses, etc. The sedge-moss zones are altogether around 12 km-wide. The final, narrow fifth zone on the valley edge consists of woodland dominated by birch and alder carr forest [74, 94–96].

### Pilot study

To define the research area, we started pilot research in summer 2017, during which villages located along the whole Biebrza River were visited. This included some randomly selected settlements and those recommended by people interviewed along the way. Eighteen villages were visited during the pilot study (Gugny, Zubole, Zucielec, Bajki Stare, Stójka, Olszowa Droga, Kołodzieje, Giełczyn, Brzostowo, Mocarze, Klimaszewnica, Goniądz, Dawidowizna, Budne, Dolistowo Stare, Wolne, Nowy Lipsk, Lipsk) and an average of three members of the local community were interviewed per village. Open, unstructured interviews were applied [97]. Middle aged or elderly people seated on benches in front of their houses were approached or traditional-looking houses were visited. If these two methods failed, the head of the village was visited. After a short introduction of the researcher, a general explanation of the research and usually some small talk, people were asked a few introductory questions, such as: *Do you live in this village? For how long have you been living here? Do/did you own meadows in the wetlands? Do you remember times when these meadows were mown with a scythe? Do you or your children still use the meadows? Do you know any other person in your village who could help answer these questions?* The first conversations with new persons were not recorded, which facilitated spontaneous and open conversations. Notes were taken during every conversation. The pilot research aimed to identify the key villages for further research—those inhabited by a sufficient number of knowledgeable informants as well as people with less extensive knowledge willing to participate in the research.

### Final study sites

**Fig. 2:**
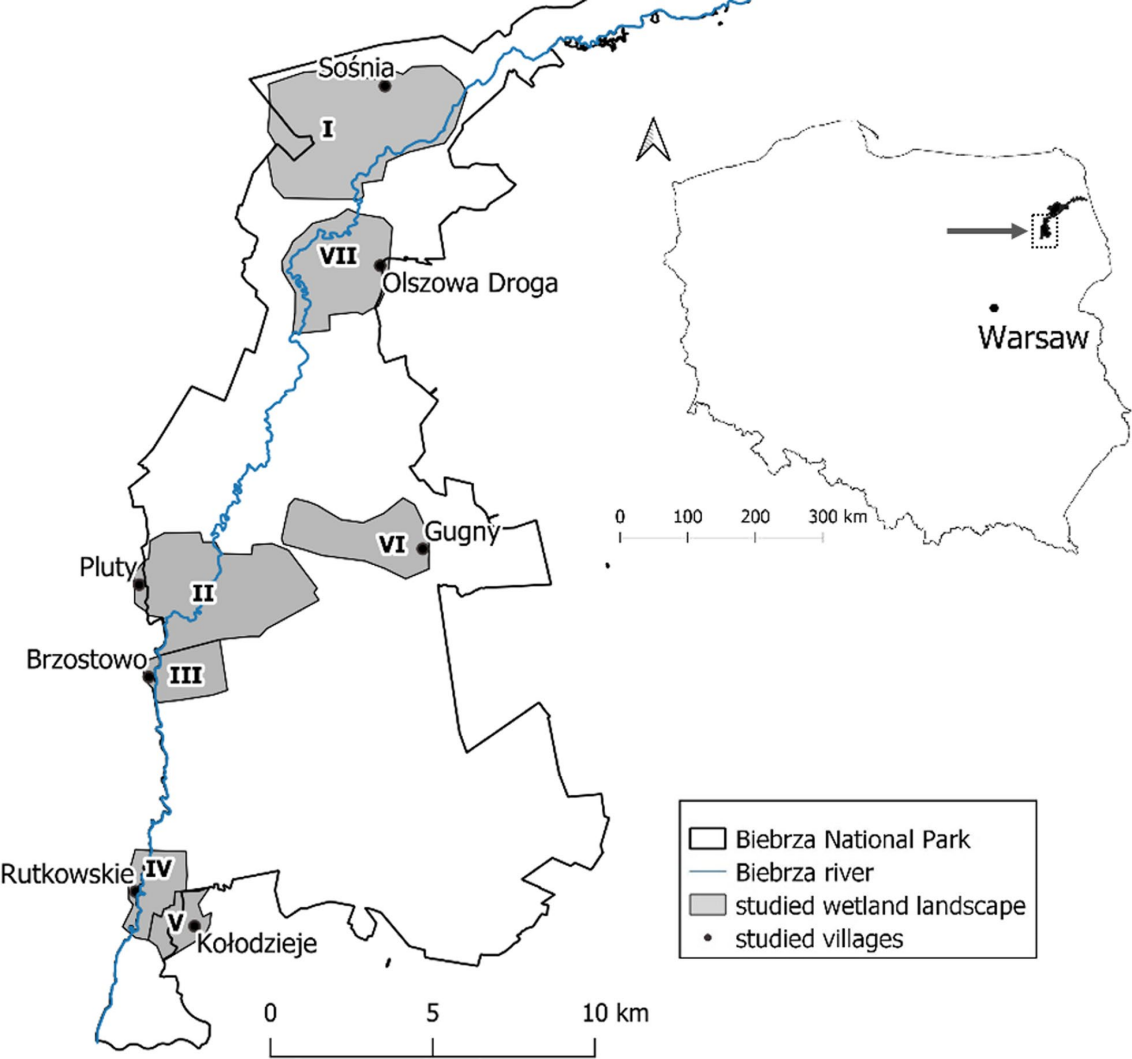
The research area of the seven studied villages in the Lower Biebrza Basin of the Biebrza National Park (Poland)

During the actual study, continued in the years 2018–2020, seven villages in LBB (Fig. 2) were selected and intensively studied using the same methods. The villages will henceforth be symbolised by Roman numerals. The villages were usually visited during summer, as the full vegetation season allowed for the application of most of the field methods. It was also noticed that people were more open and talkative in the summertime than in late autumn and winter. Each of the studied villages was inhabited by at least two local people with extensive wetland ecological knowledge. The villages are located on different sides of the Biebrza River. Four of them stand on the right side of the river, and inhabitants of these villages own meadows in the wetlands directly connected to the river. The remaining three villages stand on the left river side. One village has meadows connected to the river, and in the other two, the meadows are not adjacent to the river. The exact wetland area of study was limited to the landscape recognised by and familiar to inhabitants of the village; the area local people would tell stories about and describe with the use of toponyms; the area owned by the inhabitants of the village in times of traditional management (even if it is not owned by them right now). The studied landscape ranged from 2.5 to 18.6 km^2^ in size. People could usually recognise a wider area, which used to be explored only in wintertime. In summertime, most of the paths connecting the two sides of the wetlands were not accessible because of high water level. Only the frozen water surface made it accessible to the villages on the other side of the river, and people from different sides of the river could meet during, e.g. winter dance parties. However, the wider landscape is perceived in a more general way, and people struggled localising some of the toponyms. Therefore, it is not included in the study.

The villages also vary according to their demography and economy (Table 1). Three villages—Pluty, Brzostowo, and Rutkowskie—are the largest studied villages, with active young farmers. Two other villages (Kołodzieje, Olszowa Droga) are much smaller but also inhabited by young and active farmers. The two remaining villages, Gugny and Sośnia, are currently abandoned by farmers, but until the 1970s these villages were active and populated by several dozen families, as stated by informants.

**Table 1:** Demography of the studied villages and the interviewed local people

Village	No. of inhabitants (2011)	No. of informants (No. acc. to gender)	Age range of informants (in 2018)	No. of interviewed active farmers	No. of interviewed retired farmers
Sośnia	12	2 (0♀ + 2♂)	65–70	0	2
Pluty	136	7 (0♀ + 7♂)	40–89	4	3
Brzostowo	104	8 (2♀ + 6♂)	29–85	2	6
Rutkowskie	150	3 (1♀ + 2♂)	57–84	0	3
Kołodzieje	ca. 30	5 (3♀ + 2♂)	58–81	1	4
Gugny	5	7^a^ (1♀ + 6♂)	58–84	1	6
Olszowa Droga	30	10 (0♀ + 10♂)	30–88	4	6
In total		42 (7♀ + 35♂)		12	30

^a^The number of informants in the village of Gugny is higher than the number of inhabitants due to interviewing people who used to live in the village but had already moved out

### Data collection and analysis

The interviews were carried out with people whose families lived in the same area (the same or the neighbouring village), at least two generations back (their grandparents’ generation). Often the most knowledgeable informants had a surname similar to the name of the local village or to names from the local community mentioned by Gloger [77], which additionally affirms the local origin of the informants. Each informant was interviewed at least once. People with the greatest ethnoecological knowledge were interviewed 2–4 times, with the conversations lasting from two to three hours. The next informant in the village was usually found using the snowball method [97]. People asked to point the villager with the deepest knowledge would, as a rule, indicate the same persons—probably the most reputable individuals in the village. The conversations had the character of semi-structured interviews, launched with a few questions mentioned in the description of the ‘pilot study’, and continued with more precise questions (Table 2). For the purposes of interview dynamics, the type and the order of the questions were adjusted to the course taken by the informant, as moderated by interviewer.

**Table 2:** The sort of the questions used in the semi-structured interviews exploring people’s TEK

1. To get a general overview of life in the village and understand the informant’s perception of farm life and the changes in local agriculture:
Has life in the village changed in the last few decades? What were the main reasons for such changes? Have farming practices in the villages changed? How do you evaluate these changes? What changes influenced farming practices the most?
2. To understand the relation of people to the wetlands and their value for people:
Was it profitable to have meadows in the wetlands? Did their value change? When did you last go to the wetlands? Have you ever mown with a scythe? How much time did you used to spend in meadows in the past? What would you do there?
3. To learn about traditional farming practices in wetlands:
On what terms did people manage wetlands historically? How did the time of haymaking look in this village? How did people use wetlands, meadows and forests? What did mowing practices look like in the times of hand mowing? How did you build a haystack? When and where did people mow? Why in this particular time and place? When did people stop using the scythe in this village? For what reason? Did you continue to use your meadow after the discontinuation of hand mowing? How has the practice of mowing changed?
4. To learn about grazing practices in the wetland:
Did you have livestock? What animals did you have? Did animals graze in wetlands? Where and when did certain livestock graze? When did the grazing season start and finish? Why? Did animals graze in the wetland forests?
5. To learn what people know about plants and account for observed changes in vegetation:
Which plants grow in wetlands? How do you recognise this plant? Where does this plant grow? Is it a common plant? Did the occurrence of this plant change? What do you think, why? Is this plant good for hay? Did animals graze this plant? Which other plant species were grazed?
6. To recognise the perception of the landscape and toponyms used by people:
Where do the most valuable/the worst plants grow? Where were certain farming practices performed (like mowing, grazing, peat excavation, and fishing)? Where is your own plot located in the wetlands? Where did the mowing start, where did it finish? Where were the haystacks built? Where did you find material for building haystacks?

After the first visit in the village and a few initial conversations, during the next interview, questions were sometimes stated in a modified way, by the usage of local expressions describing elements of landscape or vegetation. If the new expression was identified, during the next interview the same person and other people in the villages were asked again for the definition of the term to ensure its meaning. All interviews were literally transcribed and 77% were digitally recorded. The collected traditional knowledge and narratives used by people (besides plant knowledge and toponyms, which were analysed separately) were grouped in a database according to topic (e.g. grazing livestock, time of grazing, mowing technique, time of mowing, haystack structure, wetland value).

**Fig. 3:**
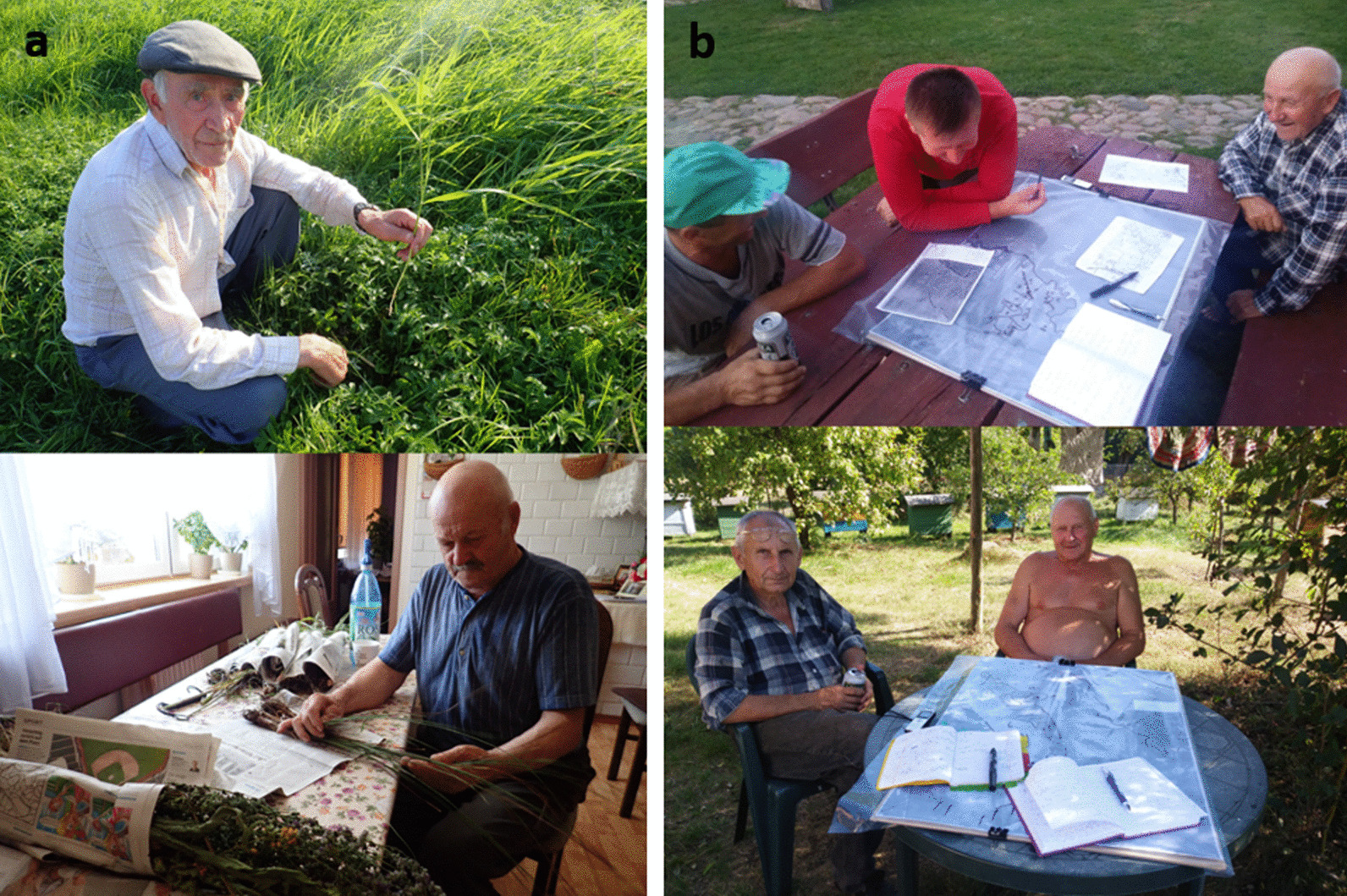
Data collection in the Biebrza Valley (Poland). **a** Plant species identification: above—walking with the informant in the wetlands, below—using fresh voucher specimens (Photos by J. Sucholas, 2020). **b** Mapping work—informants from different generations cooperate while creating the local wetland map (Photos by J. Sucholas, 2019)

To detect if people had knowledge about the plants, the questions related to plants from Table 2 (point 5), were asked. In the next step, to correctly identify ethnospecies to botanical species, one or two of the following methods were applied [97] in the case of the most knowledgeable informants (Fig. 3). First, just before the interview, fresh voucher specimens were collected in the wetlands belonging to the village; later these visual stimuli were presented to the informant. This method was applied the most often, and it was the most successful in terms of plant verification by informants. People often immediately recognised plants they could touch, smell, and see in their real size. Second, if the interview was held in the winter season, another kind of visual stimulus in the form of pictures of the wetland plant species was used. For this method, A4 size sheets with photographs of 125 vascular plants occurring in the LBB were shown to the informant. This method turned out to be the most ineffective, as it was challenging for informants to recall plants on the basis of their pictures. After a few applications, the method was no longer implemented. Finally, in every village, one guided tour in the wetlands with the most knowledgeable informant in the village was made to identify plants in the field and note for changes in vegetation observed by the informant. This method was effective in terms of informants identifying ethnospecies and sharing remarks about changes in vegetation. All the ethnospecies mentioned by informants at least twice and ecological knowledge about these taxa were set into spreadsheet. The ethnospecies were classified into lower and higher systematic domains after folk biological taxonomy and nomenclature proposed by Berlin [98] and Brown [99]. The scientific plant nomenclature and author abbreviations follow The Plant List.^1^

To understand the perception of the landscape by the local community, locate the land-use practices and learn the toponyms used in the village, people were asked questions from Table 2 (point 6) related to landscape perception. Often, the toponym interviews were group interviews with older and younger generations. The younger man (son or neighbour) assisted the older man to arrange toponyms on the map (Fig. 3). Sometimes, together with the informant, the wetland landscape and its partitioning were drawn by hand on a sheet of paper. Printed maps were used for precise localisation of certain management practices and toponyms in the landscape. For each wetland landscape of the seven studied villages, A0-size maps were printed at 1:25,000 scale. Three maps shared by the Biebrza NP were used: the topographic map from 1960, aerial pictures from the area (from times of traditional management, July 1966–1967) and the most recent orthophoto map (July 2015). For mapping toponyms, the informants could use the map they found most familiar and understandable. All information related to the map was plotted on transparent overlays. If the area was covered with too many names, number coding was used for toponyms. The landscape partitioning presented in the results, the simplified traditional land-use map (showing practices from the 1960s) and a cross section of the elevation pattern were created with Inkscape Software. We set toponyms and the information noted during interviews (e.g. explanation of the toponym’s meaning, traditional practices that took place in this location or related oral stories) in the spreadsheet. Afterwards, we clustered toponyms into the landscape element sets named by these toponyms.

## Results

### Who knows what?

The research revealed the presence of LTEK possessed by members of the local community in the Biebrza Valley, varying in type and complexity according to age, gender and personal qualities (like good memory or talkativeness). Women constituted 15% of all informants. Only elderly women were recommended as ‘good informants’ in the village. All of the women informants were above 70 years old. Their descriptions of traditional practices were very simplified and general; they were able to list a few wetland species (mean of 7 ± SD = 2.5, max. of 11). As a rule, they had a good knowledge about medicinal plants species, because they used to collect them for trade besides dealing with farming duties. The majority of the informants were men who had usually farmed their whole life. Among them were two local foresters and two beekeepers who also worked on farms of their own for at least part of their lives. We distinguish three groups of male informants according to age: 1st—older than 70 years (15 persons), 2nd—between 46 and 70 (14 persons) and 3rd—between 29 and 45 years (six persons). The informants from the oldest group had experienced wetland mowing by hand as adults. They were the major source of information about studied traditional practices, the annual cycle of hay management, agricultural transitions and the drivers behind them, and long-term changes in the landscape and vegetation (as eyewitnesses to all of these processes). In this group, two men were the most insightful informants from all the interviewed people. The younger men from the 2nd group experienced times of mowing by hand in childhood, remembered traditional farm life more from observation and oral stories transmitted by the elderly than participation. Nevertheless, all of these informants had used a scythe at some point in their lives. Frequently they were not sure about the chronological order of the historical changes in farming or the details connected to certain wetland use practices. Men from the 3rd group did not experience mowing with a scythe at all and could not remember any traditional practices. Nevertheless, all of these young farmers were taught farming by their fathers to some extent and continued to use meadows in wetlands.

Informants from the 1st group could name a mean of 14 ± SD = 9.3 plant species (max. of 36); the 2nd group could name a mean of 16 ± SD = 9.2 species (max. of 40); the 3rd group, a mean of 8 ± SD = 3.3 species (max. 13). However, the group of the oldest informants provided the most comprehensive, almost ‘palpable’ descriptions of the plants’ features, habitats and farming value, in contrast to the younger informants who, even if they listed more species, could rarely name more than two qualities of the plant. The group of youngest informants could list species, though they struggled to identify them properly.

The knowledge of toponyms was shared usually during group interviews by 2–3 persons per village. The informants were from all age groups and cooperated while mapping work. The representatives of the youngest generation were full-time farmers. Informants from the 1st and 2nd group provided all toponyms used in the village. Farmers from the youngest generation who facilitated the mapping work were usually familiar with the majority of the toponyms, but some were new to them. Informants as a rule named toponyms while describing the location of their plot or farming activity in the wetlands.

### *Formerly, Biebrza meadows were the only livelihood* (III)

People repeated that wetland meadows used to be of great farming value: *these meadows were a treasure* (…) *generally it was good hay and the cow could eat everything* (VII). Informant also added that the wetland meadows were the only type of meadows available to local people in some villages: *Who would suppose that one could have a meadow in a crop field* (not in the wetland)? *No one knew that*! (III). It was the reason behind the high economic value of wetland meadows, expressed by many other narratives: *formerly, a man had to pay big money to buy a meadow* (…) *a man would sell a crop field to buy a wetland meadow* (III); *at that time, 1 ha of a meadow had a value of 350 pigs* (…) *people had to collectively buy meadows* (VII); *the one who had meadows along the Biebrza was rich* (VII). People connected the moment of the rapid drop in the value of wetland meadows with the moment of discontinuation of scythe use: *when the scythe ‘collapsed’ then the value of the meadow dropped prospectively* (…) *we abandoned these meadows* (III). Informants also confirmed that drainage of some of the land was the main reason for the abandonment of undrained wetlands: *after melioration we didn’t use them much anymore. It drove us out of the Biebrza meadows; we stopped using them en masse* (VII). Additionally, people indicated the role of artificial fertilisers, which greatly improved the productivity of drained meadows. Indeed, in this region, in the late 1970s, the ‘Association of Agricultural Cooperatives’ (*Spółdzielnia Kółek Rolniczych, SKR*) started operating actively and influenced farming practices by e.g. convincing farmers to use mineral fertilisers: *they asked us to buy it so we used a lot of it* (V). This dramatically diminished the value of undrained wetland meadows, which were impossible to fertilise and less productive: *there is no sense in using fertilisers on the Biebrza meadows, because water will wash out everything* (II). Until the process of agricultural intensification in the Biebrza Valley, haymaking and grazing predominantly took place in wetlands.

### Three types of wetland landscape partitioning

**Fig. 4:**
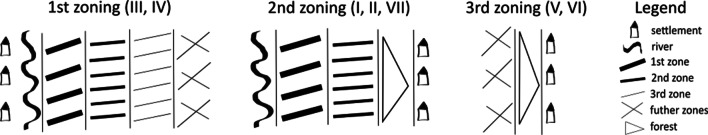
The three types of partitioning (zoning) of wetland landscape in the studied villages (Biebrza Valley, Poland) differentiated by local people. The Roman numerals in parentheses indicate the village: I—Sośnia, II—Pluty, III—Brzostowo, IV—Rutkowskie, V—Kołodzieje, VI—Gugny, VII—Olszowa Droga

People perceive the Biebrza River as the main natural border dividing the landscape and vegetation into two major areas: before the river and on the other side of the river (the latter was generally called ***zarzecze***). We identified three general types of wetland landscape partitioning present in the narratives and perception of the inhabitants of studied villages (Fig. 4). This perceptual division of wetlands into management units depends on the location of the village and roots in traditional practices; it is directly associated with the vegetation type, quality of hay, and division of land ownership.

For villages situated directly in front of the river (1st zoning) the whole managed wetlands lie on the other side of the river. Inhabitants of these villages use the term ***branie***, ‘taking’, for the zones they distinguish in the wetlands. This expression is connected with a mowing practice—‘taking hay’. The first zone is called ***pierwsze branie*** ‘first taking’; the next zone is called ***drugie branie*** ‘second taking’. This pattern continue in the further zones. In the case of these villages, ***rzeczna trawa***, ‘river grass’, which produces the best quality hay, occur in the first two zones. The ‘river grass*’* was either mown twice a year and used as pasture in autumn (IV) or the ‘first taking*’* was used for the whole season only as pasture, ***smugi***, and the ‘second taking*’* was mown once a year and mixed with grazing (III). In the 1st wetland zoning, all of the following zones, which have worse-quality hay, are called ***biele*** or ***bielne łąki***—‘white meadows*’* (III, IV): *every next taking provides a lower hay quality* (III). These meadows were mown only once a year (IV) sometimes mixed with less intensive grazing (III, IV). They are also called ***pracowite łąki*** ‘hard working meadows*’* (IV), because they are arduous to mow due to the presence of sedge tussocks. The term ‘white meadows’ is alternatively used in a wider context to name all the wetland meadows and distinguish them from any other meadows outside of the valley. The name derives from the phenomenon of fog overlaying the meadows in the mornings and evenings, which, from the perspective of the village, gives an impression of ‘white meadows’. However, in further descriptions regarding the ‘white meadow*s’*, we mean, as abovementioned, wet meadows of worse hay quality.

For the inhabitants of villages situated further from the river (2nd zoning) the wetland landscape on the two sides of the river has two major zones. The first zone of ***trawa rzeczna***, ‘river grass’, directly by the river, produces best hay quality and was mown twice a year (I) and mixed with grazing (II, VII) or mown once and mixed with grazing (II). The second zone is called ‘white meadows*’* and was mown once a year for hay mixed with grazing (I, II, VII). In this case, the forest separates settlements from meadows.

The informants from the village who own wetland without connection to the river (3rd zoning) call all wetlands belonging to them ***łąki torfowe*** ‘peat meadows’ (VI) or use the general term for worse meadows ‘white meadows’ (V, VI), as discussed before. These meadows, situated the farthest from the river, produce the worst hay quality and had different use (see Table 3).

**Table 3:** Elements of the wetland landscape of the studied villages (Biebrza Valley, Poland) and their traditional use

Element of landscape	Meaning	Traditional use	No. of toponyms/village					
			I	II	III	IV/V	VI	VII
***biele***	All wet meadows in general	Various uses	1	1	1	1	1	1
***biele, biele wytrzebowe*** (VII)	White meadows	Mown 1/y and grazed by cattle (I, II, VII); mown 1/y + grazed by cattle and horses (III, IV); mown 1/y or seldom 2/y + grazed by cattle and horses (V)	11	20	5	14	9	13
***dołek*** (VII)	Small depression near mineral islands; generally hollow in wetlands (VII)	Cattle waded there (I); cows were milked here (III); mown 2/y + grazed by cattle (VII)	3	0	1	0	0	1
***droga, grobel, drożyna*** (VI)	Road, causeway; generally unpaved, piled up roads in wetlands	Used for transporting logs to the river (I); used to get to distant meadows (I, II, IV, VI, VII); river crossing for livestock (III); used to get to the distant village (IV/V);	6	4	1	3	2	1
***grądziki***	Mineral islands; woody patches in wetlands with deciduous trees like *Quercus robur, Carpinus betulus, Pyrus* spp*.* etc	For drying of ***żaki*** (fyke nets) (I); grazed by cattle (I, II, V, VII) and horses (I); grazed in spring by cattle (II, VII); grazed ***smugi*** (III); mown 1/y (I, II, III); source of wooden material for faming tools and structures (all); source of wood for burning (II, VII)	13	7	2	4	1	8
***jeziora, bajora***	Lakes (oxbow lakes), swampy ponds	Fishing with ***niewód*** (seine) (VII), ***brodnia*** (type of drag net) (I, VI, VII), ***żaki*** (fyke nets), ***kozak*** (*a type of a fyke net with ‘hearts’ on two sides* [I]), ***kłomla*** (wooden landing net) (I, IV); soaking freshly cropped hemp (II); cleared with a scythe (I, II, III, IV, V, VII)	11	7	3	9	3	11
***krzewina***	Shrubs on wet meadows; usually applied to shrubby *Salix* spp*.* (e.g. *Salix cinerea*)	Source of material for farming tools and haystack structures (all); used for basket weaving (IV)	0	2	1	1	1	0
***kultury***	Cultivated forest fragments; freshly planted fragments of *Alnus* or *Pinus sylvestris* or *Picea abies* forest	A state property; they used to be fenced for some periods to prevent grazing	0	0	0	0	1	1
***las, grąd***	Forest, deciduous forest on hills and hollows (II,III,V)	Source of wood for burning (II, III, V); source of material for farming tools and structures (all); mown for hay (II); grazed by cattle (II, V)	0	2	3	2	3	0
***las iglasty***	*Pinus sylvestris* or *Picea abies* forest (I,V)	Source of material for farming tools and haystack structures (all); source of wood for burning (I, V, VI, VII);	2	0	0	4	1	1
***łąki uprawne***	Cultivated meadows	Generally not present during the time of traditional management but now present in the areas and in the narration of people	0	1	1	1	0	0
***łąki torfowe*** (VI)	Peat meadows	Mown 1/y + grazed by cattle and horses	0	0	0	0	1	0
***ogrody***	Gardens	Growing of vegetables (I, III, IV, V, VI); grazed by sheep (I, VI); growing of vegetables and hemp (II); growing of flax (III)	1	1	1	1	1	0
***olsyna; las olszynowy***	*Alnus glutinosa* forest	Source of material for farming tools and haystack structures (all); grazed by cattle (II) + horses (III) + pigs (VI) + sheep (VII); source of wood for burning (VI, VII)	1	2	1	2	1	1
***parowy, błota***	Muddy meadow, muds	Used as a pasture for cattle (I); mown 1/y + freely grazed (II)	1	1	0	1	1	0
***pola***	Crop fields	Arable land used to grow cereals and potatoes (all); mixed in landscape with ***smugi*** (I)	9	1	1	1	1	1
***rów, rowek***	Ditch, small ditch	Local melioration (I, II); cattle would drink water here (II); general melioration in the 19th c. (IV/V, VI); fishing with ***brodnia*** (type of drag net [VI]); cleared with a scythe (I, II, IV, V, VI)	2	5	0	2	1	0
***rzeka***	Main river course	For fishing (II, III, IV, VII)	1	1	1	2	0	1
***siedlisko***	Settlements	Farm buildings	1	3	1	3	1	1
***smugi***	Typical pastures on wet meadows or between crop fields	Used for hay making + grazed by sheep and pigs (I); grazed by horses and cattle (II, V), grazed by geese, ducks, cattle, horses, sheep, pigs (III); grazed by cattle (IV)	1	19	3	1	0	0
***torfy***	Place with peat excavation	Peat was excavated for burning (I, II)	2	2	0	0	0	0
***trawy rzeczne, łąki rzeczne, równe łąki*** (IV)	River grasses, river meadows, flat meadows; generally wet meadows directly by the river	Mown twice a year (I) + grazed by cattle (VII) + horses, sheep (IV); mown 1/y or 2/y + grazed by cattle (II); used as pasture or mown 1/y + grazed by cattle, horses (III)	3	8	1	3	1	5
		**In total**	69	87	27	55	30	46

The Roman numerals in parentheses indicate the village: I—Sośnia, II—Pluty, III—Brzostowo, IV—Rutkowskie, V—Kołodzieje, VI—Gugny, VII—Olszowa Droga. The landscapes of vil. IV and V overlap, hence the no. of toponyms are summed up

### Landscape of traditional practices

**Fig. 5:**
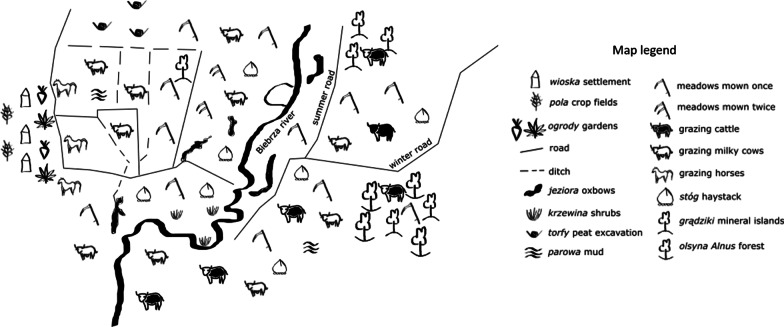
A simplified map presenting the traditional land-use of one of the villages, Pluty (II), in the Biebrza Valley, Poland, in the 1960s. Drawn by J. Sucholas. For detailed explanation of landscape elements see Table 3

The landscapes of the studied villages consist of repeating elements, structures or habitats, however, seen from different ‘village’ perspectives, their traditional function and use varied (Table 3). Figure 5 presents a simplified map illustrating the traditional landscape of the village Pluty (II), which is the most diversified among the studied villages.

The situation of this village in relation to the river is exceptional, as there is a large accessible area of usable wetlands before the river. The village stands out with the highest number of toponyms saved in the memory of the people (mainly the older informants)—87 in total (on 14.1 km^2^). Less than 18% of all toponyms refered to the area of use on the other side of the river, ***zarzecze***. The vast majority of the toponyms name elements of a landscape of an area less than 5 km^2^ in size, in front of the river. A similarly high number of 69 toponyms is used in the village Sośnia (18.6 km^2^), now abandoned by its native inhabitants. The village is also situated on the right side of the river and separated from the river by an extensive area of accessible wetlands. Both wetland landscapes (I, II) have been the most anthropogenically modified by piled up roads, digging ditches and peat excavations in former times. Fifty-five toponyms were collected in Rutkowskie and Kołodzieje, the wetland landscapes of which overlap (6.1 km^2^); then 46 in Olszowa Droga (9.2 km^2^); 30 in Gugny (5.5km^2^); and 27 in Brzostowo (3.6 km^2^). We can see that the more diversified the landscape is by the presence of many oxbow lakes, ditches, paths, mineral islands and the like, the more toponyms were invented by inhabitants. However, people underlined that: *in the past more names were in use that are forgotten now* (II). The local wetland map, rich in many toponyms, was necessary for the local community to orientate itself on the, even if modified, still broad, monotonous and flat landscape. It helped to find his or her own plot, which was not separated by any fence. Sometimes borders were marked with stones (II), small poles (III) or willow branches (IV, V).^2^ However, such a map exists only in the memory of people who never preserved their oral transmission in written form. The toponyms of meadows were derived from the surname of the owner, the name of the owner’s village, a neighbouring landscape element such as e.g. an oxbow, a distinctive landscape feature or certain farming practices. The information about times of traditional farming is encoded in toponyms. For example, toponyms indicate places of former peat excavation, owners of meadows (even if 40 or more years have passed since the land was under their management), grazing animals, growing plant species etc. Generally, even the oldest farmers admitted that they do not know the origin of a name and said that it is very old.

As a rule, the landscape element closest to settlements is ***ogród***, the ‘garden’, where people used to grow vegetables and cultivate plants for fibre, such as flax or hemp. ***Pola****,* ‘fields’—the ground on the elevated banks of the valley—were used as arable land to grow potatoes and cereals like oats, rye or millet and located around houses (I, V, VI, VII) or behind them (II, III, IV). The old riverbeds—oxbows—are a frequent element of the landscape of villages with access to the river. Notably, the landscape of wetlands near the villages from the upper LBB is dominated by oxbows marking old courses of the Biebrza River (I, VII). In times of traditional management, biomass in the oxbows and in the ditches was regularly removed with a scythe. Stories about using oxbows for fishing with traditional equipment are still vivid in the memory of the people. The term ***łąki uprawne*** ‘cultivated meadows’ is now applied to the meadows outside the valley as distinguished from ***biele*** (all wetland meadows in general). Fertilisers and seed sowing improve the productivity of cultivated meadows.

Mineral islands occur as elevated deciduous woody patches in wetlands and served many functions for people (Table 3). Grazing livestock could find shelter, shadow and fodder there. Mineral islands were the first accessible pastures in the wetlands in springtime (III, VII). The islands served as the safest place to build haystacks, secure from high water levels. The natural depression, ***dołek***, adjacent to the mineral island produced the thickest and best hay quality, dedicated especially to sheep: *hay from the hollows was deliberately separated in a haystack for sheep that ate it directly ‘in the air’* (very quickly, before hay reached the ground in the barn), *it was of such a wonderful aroma* (VII).

Predominantly, the term ***smugi*** refers to the area that is used solely as a pasture. In one village (I), ***smugi*** relates to the small meadow stripes formed in the wet depressions between stripes of elevated crop fields, not only grazed but also mown.^3^ This characteristic stripe mosaic of meadows and crop fields was situated only near the settlement (I). Pastures, ***smugi***, were either found in wetlands (II, III, V), or around settlements (all villages), or exceptionally in the area of the crop fields (I, IV). The majority of the ***smugi*** in wetlands as well as some forests were used by the whole village. Such areas were named ***ogólne***—‘communal’ (common land).

**Fig. 6:**
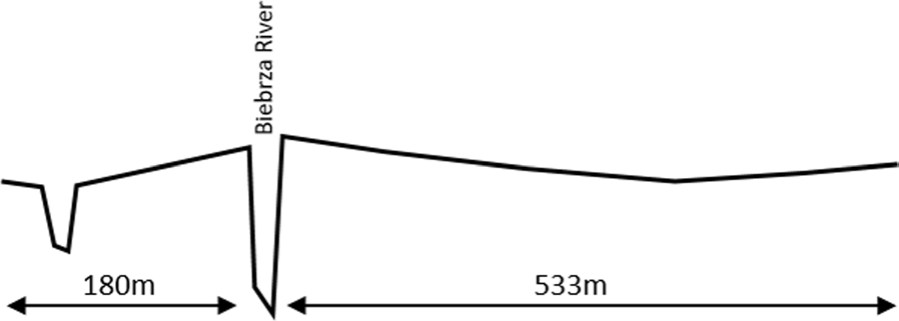
Fragment of a cross section of the elevation pattern in wetlands by the Biebrza River (based on stratigraphic cross section no. 22, Olszowa Droga village area, made in 1960s, from *Wyniki badań glebowo-florystycznych w dolinie Biebrzy dolnej* by J. Oświt, 1965 [100])

The inhabitants noticed even slight vertical drop between the river’s course and the surrounding floodplain (Fig. 6). They understand that water flooded from the river and its content work like fertilisers, thus the ‘river grasses*’*, which were flooded most intensively, have the utmost hay quality and are the most productive, in contrast to the most distant ‘peat meadows*’* that are not flooded by the river and are the most unproductive. Additionally, people differentiated plant habitats by a vertical drop—some of the plants grow in higher, others in lower wetlands e.g. in relation to the elevation to the river. The direction of the flooding water and its practical meaning in wintertime were also observed: *when the first frost came, the haystacks built on lower wetlands had to be transported to stables in the first place, otherwise there was a danger of the stacks being undermined and destroyed by the water coming quickly up-down the ice* (VII).

### Local traditional wetland management practices

**Fig. 7:**
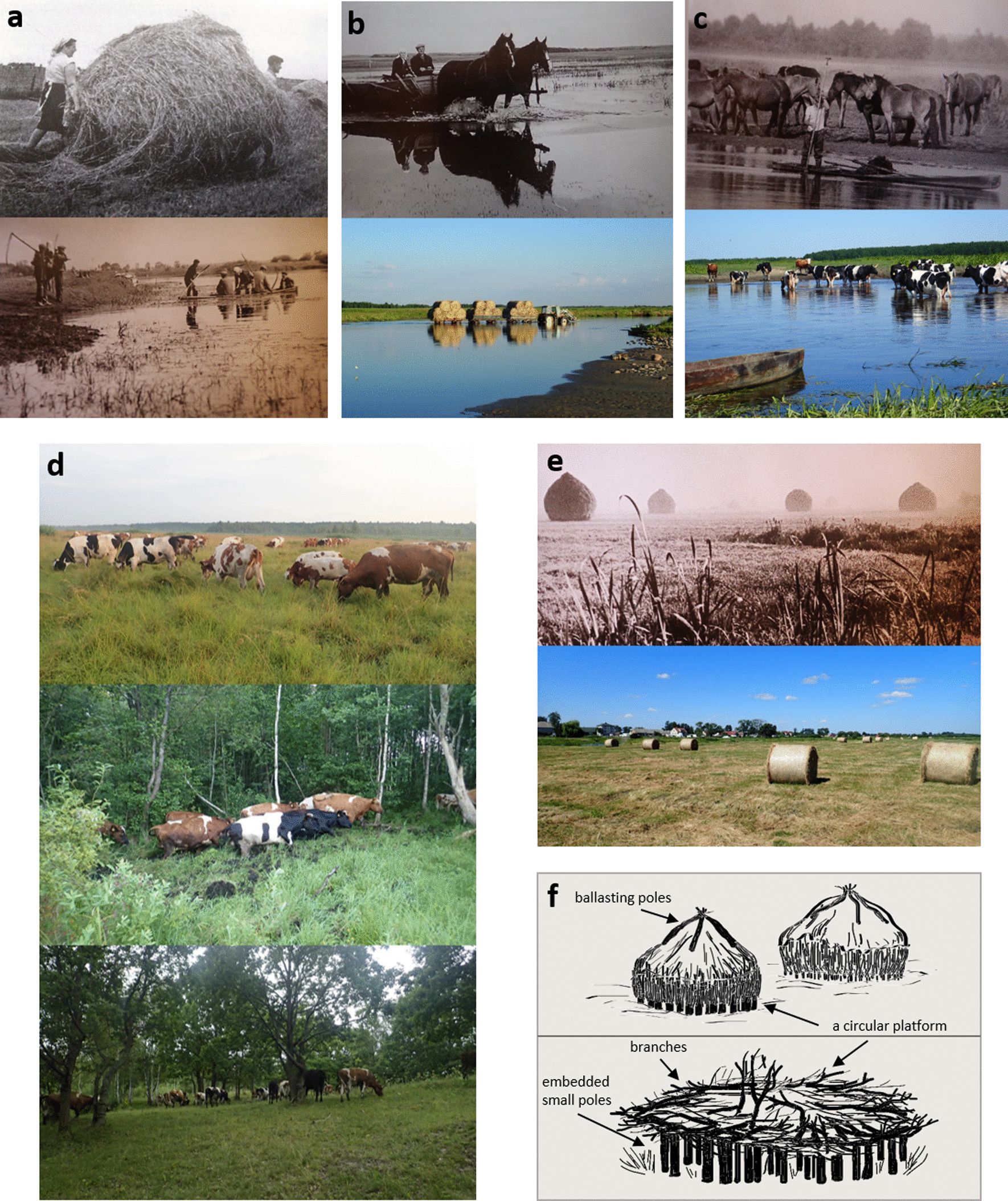
Traditional and modern wetland management in the Biebrza Valley (Poland). **a** Traditional hand hay management: above—carrying small haystack on wooden spruce rods (Photo by M. Pokropek, 1968), below—preparing to mow with a scythe (Photo by J. Rybiński, 1960s). **b** Transport across the river: above—with horses (Photo by J. Rybiński, 1960s), below—haybales transported with a tractor (Photo by J. Sucholas, 2019). **c** Livestock grazing wetlands on the other side of the river: above—horses (Photo by W. Wołkow, 1960s), below—cattle (Photo by J. Sucholas, 2018), **d** cattle grazing three types of habitats: uppermost—open wetlands, middle—*Alnus* forest, below—*Quercus* forest on mineral islands (Photos by J. Sucholas, 2018), **e** hay storing: above—traditional haystacks in the wetlands (Photo by J. Rybiński, 1960s), below—modern bales (Photo by J. Sucholas, 2019), **f** traditional haystacks on Biebrza wetlands: above—haystacks, below: empty haystack platform (redrawn after Denis Clavreul from *Portrait of a Living Marsh* by Robin D’Arcy Shillcock, 1993 [101])

The annual cycle of hay management practices in the wetlands was conditioned by natural factors, of which water level was the most important: *Everything depended on water* (all villages) (see Fig. 7). Open areas and forest in wetlands were the major habitats used to feed the livestock, especially cattle and horses, but also pigs, sheep, geese and ducks. *In the early spring, when water moved back from the wetlands and the first grass was visible, cattle was released for grazing on mineral islands* (II) or *since the beginning of May milk cows grazed there* (on mineral islands) *the whole month, day and night (…) twice a day we went there by boats for milking* (VII) (Table 4). Since the pastures in the open wetlands had no fences, livestock either grazed freely in large areas or all cattle belonging to a village was herded by one person, sometimes even a child, in turn: ***kolejka; wypas kolejką,*** ‘queue’. According to this method, the owners of the cattle tended them one by one, for a number of days equal to the number of owned cattle or half of this number (all villages). The fowl (geese, ducks) was usually kept in small pastures around the settlements, besides one village with easy access to mineral islands on the other side of the river (III). This area was used as pasture, ***smugi***, for various livestock species grazing altogether (Table 4). The situation was similar when it came to pigs, which foraged near the houses, with the exception of the abovementioned village, where they were fed in ‘river grasses’ (III) and villages (VI, VII) where pigs would graze freely in the *Alnus* forest nearby the settlements. A few informants said that cattle used to eat saplings in spring (VI, VII), which occasionally had ill effects: *everyone had to have vinegar at home to cure a cow which used to have blood in urine after eating saplings of the lime tree* (VI)*.* Sheep were fond of grazing wetland meadows, however, they required drier ground and had to be in sight of people to protect them from wolves. Because of this, they grazed close to the settlements: in drier ***smugi*** (I), ‘river grasses’ (III, IV) or *Alnus* forest and ***biele*** in autumn time (VII). Interestingly, in the past, one of the villages (IV) already had pastures in the zone of crop fields, which were grazed by cattle, horses and sheep until the last mowing date in the wetlands. After the second mowing in the wetlands, the livestock could freely graze ‘river grasses’ belonging to this village. Horses ordinarily grazed freely in the wetland meadows and forests at night, since during the day they were needed at the farm: *horses made groups while walking in wetlands in the night to protect themselves from the wolves* (VI). Sometimes catching the scattered horses in the morning was challenging, so *the horses were herded to the heaviest mud to hogtie them* (VI). In one of the villages (II) they grazed in wetland pastures situated very close to settlements during the day. The following four types of cattle grazing regimes in wetlands are noted: (1) if the wetlands were intended for hay making and had to produce grass, the cattle grazed in forests during this period, usually tended by the owners in turn using the already described ***kolejka*** system (V, VI, VII); (2) they grazed pastures close to settlements until the last mowing in wetlands (IV); (3) in the case of large wetland areas, heifers were herded to the ***biele*** in early spring and grazed freely day and night until autumn (I, II, III); (4) the lactating cows could graze ***biele*** destined for mowing under the herder’s supervision (I, II, III). The lactating cows were either visited twice a day and milked in wetlands or milked every evening once back home. Many informants believe that cows had less milk in their udders after grazing wetlands, because it was ‘sucked out’ by the snakes. Traditionally, livestock grazed in wetlands until first snow—***do zapadłych****—*as confirmed by all informants.

**Table 4:** Time and place of traditional farming practices such as mowing and grazing in the Biebrza Valley

Traditional practice		When	Where
Mowing	First	Around St. Peter's Day 29th of June (II, III, V, VI, VII), around St. John's Day 24th of June (I, IV)	***biele*** (V) + river grasses (I, II, III, IV, VII); peat meadows (VI); ***smugi*** (I); mineral islands (all); ***grąd*** (II); depressions (VII)
	Second; called ***Otawa*** or ***Potraw***	End of August/beginning of September (I, II, IV, V), around The Nativity of the Blessed Virgin Mary 8th of September (VII)	River grasses (I, IV, VII); river grasses in front of the river (II); depressions (VII)
Grazing	Cattle	April/May—15.10/01.11	***smugi*** + ***biele*** + mineral islands (II) + *Alnus* forest (III); ***grąd*** (V); *Alnus* forest (VI)
		15.05–01.11	***parowy*** + ***biele*** (I)
		In May, before 1st mowing	Mineral islands (VII)
		During mowing time	*Alnus* forest (VII)
		After 1st mowing	***biele*** (V, VII); peat meadows (VI)
		After 2nd mowing	River grasses in front of the river (II); river grasses (IV, VII)
	Horses	1.05–15.10	***smugi*** + *Alnus* forest (II) + ***biele*** + mineral islands (III); ***grąd*** (V); peat meadows + *Alnus* forest (VI); river grasses + ***biele*** + mineral islands (VII)
		15.05–01.11	Mineral islands + ***biele*** (I)
		After 1st mowing	***biele*** (V)
		After 2nd mowing	River grasses (IV)
	Sheep	1.05–15.10	***smugi*** (III)
		15.05–01.11	Mineral islands + ***smugi*** (I)
		After 2nd mowing	river grasses (IV)
		Autumn	*Alnus* forest + ***biele*** (VII)
	Pigs	1.05–15.10	***smugi*** (III); *Alnus* forest (VI, VII)
		15.05–01.11	***smugi*** (I)
	Geese and ducks	1.05–15.10	***smugi*** (III)

The Roman numerals in parentheses indicate the village: I—Sośnia, II—Pluty, III—Brzostowo, IV—Rutkowskie, V—Kołodzieje, VI—Gugny, VII—Olszowa Droga

All informants admitted that the time of the first mowing depended on the water level: *grass started to grow at the end of May* (…) *usually, first mowing was at the end of June* (…) *but it depended on the water level* (VII) (Table 4). However, as a rule, the Catholic feast days, e.g. St. Peter's Day 29th of June and the Nativity of the Blessed Virgin Mary 8th of September, fixed the start of the first and second mowing respectively, which would begin one day after them. However, if the water level was still high, the first mowing could be delayed until July. At the time of traditional management, all open wetlands and grass in deciduous woody patches were mown: *formerly, with a scythe, everything was mown as low as possible* (…) *with a scythe one could enter everyplace, even if the water was still there* (VII). The time and place of the second mowing was conditioned by the water level and type of vegetation. Nevertheless, grass needed to be cut at the beginning of September at the latest: *if hay was cut later, cattle did not want to eat such hay* (VII). Sporadically, intensive rainfall in summer prevented the second mowing. All the ‘river grasses’ which were not used as pastures were mown a second time. A few informants stated that the second cut provided even better hay quality than the first cut: *first hay was a very good fodder—out of thicker grass, however, the second was better—out of very soft grass* (IV). If ‘river grasses’ were not available to a community, but only the worse quality ‘white meadows’, then these were also mown once or occasionally twice (V). Mowing in wetlands usually started from the side of the river and was performed by the whole village simultaneously. It engaged people for intensive 2-week-long work.

Natural conditions shaped the development of haymaking methods and defined the used materials. All informants said that depending on grass quality, it was mown either ***na pokos***—‘in swath’ (in case of thicker grasses, the haymaker would mow a swath only from one side—mowing always in one direction) or ***na zbijaka***—‘to conglomerate’ (a technique applied to thin grasses; the haymaker would mow grass from two sides to achieve a thicker swath—mowing in two directions, back and forth). The first technique was the predominant, while the second was used on very poor-quality ‘white meadows’ (V, VII); grasses in the most distant ‘taking’, like fifth and further zones (III); or on ‘peat meadows’ (VI). *It was good to start mowing at dawn with the dew when grasses were fresh and not dried by sun* (VII). During dry and sunny weather, hay did not require turning. When hay dried out, women raked it and formed ***kopy*** (small haystacks). Usually, a couple of people would bring these small haystacks to the final large haystack on ***nosidła*** (two special wooden rods around three metres long used like a stretcher). Sporadically, hay was not carried by a couple of people but situated with a rope on one wooden pole that was attached to a horse which then carried it to the stack.

A haystack was preferably built on a mineral island if it occurred in the owned plot of meadow, or, as was usually the case, in the middle of the plot (as the most easily reachable and optimal location). As only the haystacks from the meadows close to the settlement were transported on a boat to stable but in most cases stayed in wetlands for the next few months until the winter, a special platform was built under the stack to prevent hay from being flooded and decaying. A few expressions were used for these platforms, such as ***art*** (VI, VII), ***hart*** (VI), ***podzisko*** (II, III, IV, V), ***łożysko*** (I, V). The structure had a circular shape. It was constructed from any available material—either stones (II, IV) or embedded wooden small poles, ca. 50 cm in height. These poles were additionally covered with branches (see Fig. 7). In wintertime, when frost made the meadows accessible, people would bring available material needed for platform structures, such as sand (IV), stones (II, IV) or branches (VII): *in summer, it was challenging for us to bring all these branches when the water was everywhere* (VII). The platform structures were so high that *when a man had to come to a stack with a boat (because water was so high) the hay was still not touching the water surface* (IV). The final stack was about 4 m high and 4 m in diameter. It was built by two people who, by giving to a stack a proper shape and arrangement of external hay, provided it with the necessary stability and waterproofing. In the LBB, all haystacks were ballasted by four heavy poles interposed on top of the whole structure. These poles had various names such as ***gręzidła*** (II, III), ***koźliny*** (IV), ***chlusty*** (V, VI, VII), ***zimówki*** (I). Various tree and shrubs species were used as material, depending on what was available (Table 5). If wood was not available, they were replaced by ropes made out of hay. As a rule, hay was transported from stacks to the stable on a horse-drawn sleigh in winter, when the wetlands were frozen.

**Table 5:** Ethnospecies of Biebrza wetlands and related local traditional ecological knowledge (contains mainly direct quotes from informants)

Life form	Generic taxa	Literal translation of folk names	Scientific name	Habitat	Localisation	Features	Mowing value	Grazing and hay value	Plant changes	Other uses
*trawy* 'grasses'	*rzeżucha* (all) *rezucha* (all) *rzeź* (VII) *rodzina rzeżuchowatych* (VII)	Bittercress Bittercress-like 'Cutting' bittercress family	*Carex* spp., e.g. *Carex elata* All.*, Carex acuta* L.*, Carex acutiformis* Ehrh.*, Carex riparia* Curtis	Where *rzeżucha* grew, there was only one mowing; it does not grow in 'river meadows' (VII); tends to grow in higher places (II); grows in lowered places in front of mineral islands (VII); a fine type grows on mineral islands covered by deciduous trees (II) and rarely closer to the river (IV); forms worse, 'white meadows' *biele* (III, IV, V); grows where the meadow is not used any more (V)	Grows halfway between forest and river (VII); small *rzeżucha* grows closer to the river, on the other side of the river there is plenty of the tall *rzeżucha* (II); it grows in the 2nd zone; in all further zones 'horse-like grass' *końska trawa* grows, meaning thicker *rzeżucha* grazed by horses (IV); on two sides of the melioration ditch (V)	It usually forms tussocks or seldom grows flat (II, VII); it is sharp on the edges, one can split one's finger when it is in the hay (II, III, IV, V, VI, VII); one can easily go over it with one's hand in one direction, in the opposite it cuts the hand open, it has little teeth (IV); it can be low, acidic (II) or tall grass, even up to 0.5 m (II, III); it can be broad and even 1 m tall (IV); lightweight (VII); there is a few species of it (VII);	When it forms tussocks it is difficult to mow; when it would fall down between tussocks it was difficult to rake by hand; it is easy to cut with a scythe (VII)	Young grass is eaten by cattle but it is left when old, hard and dry (IV, VII); cattle eat only the tips of the old grass; generally livestock was not keen to eat it (V, VII); it is 'horse-like grass'—horses are willing to eat it (II, IV); the old and dry has no value (VII); it can be used for bedding or as fodder—it cleans the bodies of cattle and acts like a buffer in fodder (II)	Today the only plants that grow are those that can break through the reed bed, like *rzeżucha* (VII); when we mowed with a scythe it formed tussocks, later tractors and machines destroyed it and the grass is different now (II)	
	*siwucha* *siwuchowate* (VII)	Greyish one Greyish ones	*Carex panicea* L.*, Carex flava* L.*, Carex nigra* (L.) Reichard	It does not grow alone but is mixed with other grasses; grows in the lowermost area; just in front of the forest where water stays all the time (VII)	Grows on the edges of the meadows, just by the forest (VII)	It has grey leaves, a little broad; it is different than *rzeżucha—*it is a rather low and softer grass (VII)	Sometimes it was so thin that it was mown from both sites to obtain a good, thick swath (the method *na zbijaka*) (VII)	It is good for cattle; cattle on meadow would eat it immediately; cattle would eat this grass instead of *rzeżucha*; it does not have too much value and protein (VII)		
	*tymotka* (VII)	Timothy	*Carex nigra* with inflorescences/ seeds	The lowest area, where water stays all the time (VII)	Just in front of the forest (VII)	It is from *siwuchowate* family but has thick seeds halfway up the plant; when one touches it the single seeds fall down (VII)		Cattle like to eat it (VII)		
	*trzcina* (II, III, IV,V,VII) *trzcinka* (VI, VII)	Reed Small reed	*Phragmites australis* (Cav.) Trin. ex Steud	Edges of the lakes; in grass where the area is lowered (III); by the river (VI, VII); in old river beds that are overgrown (VII)	By the lakes (III) and the river (VI, VII) and in overgrown oxbows (VII)	It grows tall and has broad leaves (IV)		Cattle like to eat the young shoots, especially young leaves (IV, VII); in spring cattle do not let it grow because it eats everything immediately (VII); it is sweet like corn (VII); cattle eat it happily because it is sweet (IV); when it is older and thick like a finger cattle do not eat it (IV); it is good grass for fodder (VI)	Formerly there was no reed by the river because cattle trampled it out; where was *rzeżucha* in the past, but now it is not mown, the reed came in; formerly there was just a little of the reed, now it covers hectars (VII); it is moved by the water flow from one place to another (IV)	
	*jęczmianka* (I, VI, VII) *jemioła* (II, III, IV, V) *niemioła* (II, III, IV) *trzcinówka* (III)	Barley-like Mistletoe Mistletoe-like Reed-like	*Phalaris arundinacea* L	Grows in the 'river meadows' (II, III, VI); on higher mineral islands (VI, VII); generally in higher places in wetlands (II, VII); by the river, where is red sand and two mowings in the year (VII); it dominates in the second mowing (VII)	Grows in the 2nd zone, on the other side of the river (III, IV); or in the 1st zone and on small mineral islands (II, VI, VII); grows between mineral islands and river, not by forest (VII)	It is tall (II, III, IV, VII); has broad leaves that come apart on the sides (III, IV); similar to reed; main stem is hard like a straw and thick like cereal; it does not grow densely (VII); softer than reed and a little twisted (II); it grows tall like rye, up to 1 m (IV); is massive and hard (I, IV, V); blossoming it has a raceme, little seeds like groat (I)	Extremely difficult to mow (I, III, VII); man needs a really good scythe to cut it (I); if somebody was too slow then one could not cut it (VII); it gives a really long swath, long like a scythe (VII);	It is not the best but not the worst grass (III, VII); it is not so edible (III); cattle eat leaves very happily, so probably it is sweet but neither calf nor mature cattle eat the hard stems; cattle eats it when there is nothing else to eat; the old people always said that it is a sweet grass and horses eat it happily; when one brought it to horse for a night, in the morning one could find only little remnants (VIII); the old, 1 m tall grass is too old for cattle; it gives better hay when it is mown early (IV); cattle likes it (IV, V); it is good grass for fodder (VI), but worse than *bluszcz* (VII)	Formerly, this grass was very rare, now it has replaced *bluszcz* (III); it appeared after melioration (VI)	
	*mózga* (all)	Might derive from verb 'to touch fleetingly' or from Polish *mozga*, botanical name of reed canary grass	Not blossoming parts/stage of *Agrostis stolonifera* L.*, Glyceria fluitans* (L.) R.Br.*, Alopecurus geniculatus* L	Lowered places, in hollows in wetlands (I, II, III, IV, VI, VII); it dominates in a second swath *na potraw,* it is rare in the hay from the fist mowing (I, III); it likes to grow at waterlogged conditions; in hollows between mineral islands (VII); in 'river grasses' (I, II, IV, VII)	It grows in the 1st zone (III, IV); by the river (I, VII) and by mineral islands (VII)	Has pointed leaves (III); it is soft, dense, fine and small grass (I, III, IV, V, VII); one cannot cut oneself with this grass; when it grows higher it lies down immediately, it cannot stay straight, because it is thin at the bottom and has broader leaves higher up that go sideways; there is no main stem, leaves grow directly out of the ground (IV); it has a nice scent (III, IV); it is a little darker grass (VI); it is not blossoming; leaves are broader than *okrąglica*; one can walk barefoot on it; when one squeezes it up, it is gone, it is like a foam (II); one walks on it like on an eiderdown (I, IV); one can sleep on it (IV); when it dries out it becomes cyanic (VII)	It is challenging to mow it because it is so soft; the mower just takes and mills it instead of cutting (VII); a scythe must be sharpened, peened, otherwise the grass just lies down (I,IV)	Cattle eat it happily (III, V, VII); formerly, when we pastured pigs, they loved it (III); it is highly good fodder grass (VI, VII); horses like it most (VII); basically, all animals liked it (I, VII), even rabbits, because it is tasty and probably also has some special properties (I); it is something good!; it is a noble grass; cattle loves it because it is fatty and the most valuable (II); when it was in the hay, everyone gave it directly to little calves; it is good fresh and dried (IV); it is the best grass of them all (IV, V)	There is much less of it because it is drier now (I, II)	
	*okrąglica* (II, III, IV, VII)	Rounded one	*Carex appropinquata* Schumach.*, Carex diandra* Schrank	It grows in worse quality meadows (III, VII); it grows in 'peat meadows'; in the place where there is no body of water, no lake, no river (VII); it is in the higher places (II)	It grows in further zones (III); it grows in meadows belonging to the village VI; it grows far away from the river, by the forest (VII); it does not grow in our meadows (II)	It usually grows in tussocks (III, VII); it is a tall grass; has rounded leaves; more rounded than *rzeżucha* (III); the stems are extremely hard; they are so hard that they shine; the stem is very thin, similar to reed but thinner; when covered with water it turns red; it is generally red; it has very less glue inside (VII); the stem is rounded (II); the stem is rounded at the bottom; it belongs to the same group of species as *miotlica*; it is very hard; it is 0.5 m tall or more and on the top has very fine, single ears; its thin stems lie down sideways (IV)	One mows this grass a bit later because it grows slowly; especially when it is grazed it has little growth; difficult species to mow; it is so hard that a scythe just lies it down or it is humming on its stem and cannot go through it; moreover when it is mown, a swath goes between tussocks and it is difficult to take it out with a rake; rakes would lose teeth during this work; usually it is mown on two sites (*na zbijaka*) to give a thicker swath (VII)	People mowed it for hay because they had no choice; it is low quality, weak grass; now they mow it just for bedding because it is not suitable for fodder; in autumn they gave it to heifers because milky cows did not want to eat it (VII)	*I cannot find it on our meadows… Maybe it changed so much? The one I see here now, is much softer* (VII)	
	*mietlica* (III), *miotlica* (II, IV) *miotła* (II, III) *miotełka* (III, V) *jęczmianka* (III)	Broom-like Broom-like Broom-like Little broom Barley-like	Blossoming stage of *Poa palustris* L.*, Deschampsia flexuosa* (L.) Trin. and other thin *Poaceae* species in wetlands	One can find it also in the cereal crop field *polna mietlica* 'crop field mietlica' (*Apera spica-venti* (L.) P.Beauv.) when it is not artificially fertilised (III); it prefers higher places (V)		It is tall and thin (II); it has dense, fine leaves and a long stem with ears; when it starts fruiting, the seeds will be everywhere (IV); fine grass (V)		Cattle like it (III, IV)	In front of the forest there should *okrąglica* grow but now grass is another, much thinner (*Deschampsia flexuosa*); *has okrąglica changed so much*? (VII)	
	*hoszczka* (all) *hoscka* (III)	Of more onomatopoeic derivation naming something that is crackling, creaking	*Equisetum fluviatile* L. (syn. *Equisetum limosum* L.), *Equisetum palustre* L	Muddy places, lowered places (I, II, III, VII); in 'river meadows' (VI); it can grow by the river (V) only when water stays longer and there is a good soil (VII); more often it is in hollows between mineral islands (VII); it accompanies *Menyanthes trifoliata* (II); it is everywhere in the meadows; here more, over there less, but everywhere in our meadows in general (IV); in watery meadows (V); it likes when there is water (I); there is also *hoszczka polna* 'crop field *hoszczka*' (*Equisetum arvense* L.), which is similar but does not grow in wetlands (III, VII)	Grows in *parowy* (area with muddy meadows) (III); closer to the river (II, VI); in muddy places on *smugi* (pastures) (II); it grows in the middle between the river and forest zone (VII); everywhere in wet meadows (IV); grows on two sides of the melioration ditch (V)	It is extremely fragile; has no glue inside (VII); it is easy to break, because the steam is divided in pieces (II); it is rounded; grows tall; the stem has 'knees', 'connectors' and when grass is dry it breaks in the knees; it is pipe-like (IV); it makes a 'snapping' sound (I); it can be even up to 1 m tall (II); one can find a similar type in the crop field (I, IV), but the river type of *hoszczka* is thicker and taller (IV)	It is not suitable for mowing (III); it is easy to mow; we could mow it with a scythe during a day (I, VII); when it dries out, then it is extremely fragile; raking had to be done in the early morning 'with the dew' or in the evening when it is softer and not so fragile; when it is dry one cannot rake it and put it on the haystack (I, II, IV, V, VI, VII); it could be raked and brought to a stack also on a humid day (IV); because of lack of the glue it would 'escape' from the rake; we had to carry small haystacks, *kopki*, with a *hoszczka,* to a haystack, supported by four pairs of forks, otherwise it escaped (VII); hay containing only *hoszczka* would be very difficult for a bale maker to collect (II)	Cattle eat *hoszczka* from wetlands but the one from the crop fields they do not even touch; but generally it is not the best grass (II); it is a tasty grass; cattle and sheep like it, but horses less (I); it is good fodder grass (VI); cattle ate hay with it, but when it was prepared in good way—sometimes when it was in a haystack in too wet form and was pressed too much, it got mouldy, so cattle did not want to eat it (IV)	Formerly, there used to be more of it (II); now there is much less of it because it is too dry (I)	It is a medicinal plant, collected and sold (II, III); only *hoszczka* from the crop field is a medicinal plant, from it wet meadows is not (IV)
	*bluszcz* (all) *blusc* (III) *miecz* (VII) *trawa mieczowata* (VII)	Ivy Ivy Sword Sword-like grass	*Glyceria maxima* (Hartm.) Holmb	Lowered places, hollows (II, IV, VII); it grows in places with waterlogged conditions; it sometimes grows between *mózga* (II) in river meadows (II, III) but also accompanies *jemioła* (IV); in muddy places, but not everywhere (V)	It grows in the 2nd zone, on the other side of the river (III, IV); it grows in the 1st zone (II); grows in the middle place, halfway between forest and river, but closer to the river (VII)	It is tall (III, VII); has broad leaves like two fingers; grows dense; it is hard; they called it fern because it has dense flowers on one side (III); the broad leaves are like feathers on two sides (IV, VII); it is from the sword-like grass family *mieczowata*; it has sweet glue inside stem; it has a nice scent; it is very sharp, rough; one can cut his fingers; it is sharper than *rzeżucha*; similar to reed but smaller (VII); it is a thick grass (IV, VII); it has broader leaves than *jemioła* and is shorter; when it is young it stays more straight, the older lies down on the ground; it bends to its side like wheat or rye; it twines, creeps; when one straightens it up, it is long; when *jemioła* grows on the side *bluszcz* vines on *jemioła*(IV)	It is demanding to mow it because it is hard (III); it is generally easy to mow, any scythe could cut it; but it takes time to dry it out because it has so much glue inside (VII); it is not easy to mow, when it lies down and somebody is not a good mower then they just touch it on surface and the whole mass stays unmown (IV)	Because it is sweet, cattle likes it (II, III, VII); it is a very good fodder grass; cattle and horses and other animals eat it happily (II, V, VII); especially young plants are a delicacy for cattle (III, IV); the old form is too hard for cattle (IV); all sword-like grasses *mieczowate* are good fodder (VII); when it ferments in bales it becomes yellow and is excellent; especially, one can give this grass to a cow when the cow is not giving milk (VII)	Now there is much less of this grass (II); formerly, there was more *bluszcz*, now it is replaced by *jemioła* (III); formerly it was rare, because it was mown every year; now there is more of it, because one cuts the grass higher (with machines) (VII); now it is more common than in former times (I)	
	*tatarak* (all) *trawa mieczowata* (VII)	Sweet flag Sword-like grass	*Acorus calamus* L	On the edges of the river (III, VII); mainly by the lakes (IV, VII); it grows by bodies of water in wetlands and generally everywhere in wetlands (VII); in more wet, muddy places, hollows, in waterlogged conditions, not on mineral islands (IV)	By the river (III, VII) and lakes (VII, IV), in the 1st zone (III)	Has broad leaves (II, III); has smelly roots (II); leaves are flat (II); it has a characteristic, thick rhizome; sword-like family grass (VII)		Cattle prefer not to eat it (IV, VII)		Medicinal plant; rhizomes are collected for sale (II, III, IV); leaves put under bread to keep it fresh (II, VII)
	*kosak* *kosac* (VII) *kosaciec* (IV) *trawa mieczowata* (VII)	*kosaciec* is a Polish name of *Iris*, *kosa* means a scythe sword-like grass	*Iris pseudacorus* L	On muddy edges of the lakes and river (IV)	By the lakes and river (IV)	When it is ripe, it has a pod similar to the broad bean (III); it blossoms yellow; sword-like family grass (VII)		It is an exception when cattle eat it (VII)		
	*sitorz* (III) *sitnik* (VI, VII) *sitarz* (VII)	*sit* in Polish means rush, all the local names are variations of *sit*	*Juncus conglomeratus* L. (III, VI, VII), *Juncus articulatus* L. (III)	Grows in acidic soils, in acidic meadows; where it grows, the grass has no value; grew in front of the forest, where cattle used to graze (VII)	Used to grow ca. 100 m in front of the forest, where cows used to graze, near the *grobel* (causeway) (VII)	It is thin and has seeds at the top; grows in groups (VII)		This grass has no value; cattle do not like it; a cow will not take it in its mouth (VII) w	When cattle grazed it, there used to be a lot of it, now there is much less of it and generally it is overgrown by reed (VII)	
	*sitorz* *sitkorz* (III) *scypiorek* (III, IV)	*sit* in Polish means rush, all the local names are variations of *sit*, chive-like	*Eleocharis palustris* (L.) Roem. & Schult	It grows between other grasses in meadows; on the elevated mineral islands (III)	Mineral islands (III)	It is very small and thin (III)				
	*sitarz* (IV)	*sit* in Polish means rush, all the local names are variations of *sit*	*Schoenoplectus lacustris* L. (Palla)							It was used for swimming lessons. One collected two handfuls of long stems and tied each of them up with a string at the ends. Then one clenched them so strongly that one got two bolsters. They would not soak with water. Then one connected the two bolsters with a strings. One could lie down on this strings and had bolsters on two sides. Then one could swim easily
*zioła* 'grerb' (+ *Lythrum salicaria L.*)	*bociany* (III)	Storks	*Lysimachia vulgaris* L.*, Symphytum officinale* L			Plants from herb family that are visible above the grasses (III)		They have no fodder value (III)		
	*żywokost* (III, VII)	Comfrey	*Symphytum officinale*	Grows on the edges of waterbodies, like river, lakes (III, VII)		Has long, broad leaves; it blossoms pinkish; has brown roots; belongs to the herb family (VII)		It has no fodder value (III, VII)		Medicinal plant (III, IV, VII); it is good for joint pain (VII); I collected it in the winter, digged it out with an axe, cut the roots, dried it out and made an ointment with oil (VII); my mother told me that people harvested it barefoot and directly put it on aching places (III)
	*tabuła* (IV, VII)	*Spiraea*	*Filipendula ulmaria* (L.) Maxim	Grows in little elevated places and on mineral islands (VII); it grows in a muddy *olsyna* (*Alnus*) forest (IV)		It blossoms white; it is tall; has quite a hard and thick stem; belongs to the herb family (VII)		It has no fodder value		Medicinal plant; people collect and sell it; only flowers are harvested; it can be cut with a sickle (VII)
	*drabinka* (V, VII) *srebrnik* (III) *gęsie łapki* (IV)	A little ladder Silver-like Little goose paws	*Potentilla anserina* L	It grows, though not neccesarily, in wet meadows; it grows close to the river (IV)	It grows in the 1st zone; it does not really grow in the further zones (III, IV)	It looks like a small ladder; has a nice scent; belongs to the herb family (VII)		Cattle eat it		Medicinal plant, people collect it and sell
	*gęsie łapki* (III, VII)	Little goose paws	*Comarum palustre* L	It grows in wet meadows, on the other side of the river (III, IV); it grows at waterlogged conditions; on the edges of lakes (VII)	It grows in further zones, closer to the forest (III, VII)	The stem creeps on the ground; one can stumble against it and fall over (IV)		It is more like weed; it is not a good fodder; it is not edible (VII); cattle do not eat it (IV)		
	*mięta* (all)	Mint	*Mentha aquatica* L	In wet meadows, everywhere (all)		It is from the herb family (VII)				
*kacaki* (+ *Alisma plantago-aquatica L.*, *Sagittaria sagittifolia* L. [III,IV])	*bobrek* (II, III, IV) *bobik* (I, II, III, IV, VI, VII) *gęsie łapy* (III) *boberek* (II) *bober* (II) *bobownik* (V)	Bogbean A little bean Goose paws Bog-bean-like Beaver-like Bog-bean-like	*Menyanthes trifoliata* L	It grows in waterlogged conditions; it must be in water (II, IV); in lowered places, in hollows (IV, V); between the tussocks (IV); in hollows in front of the mineral islands, together with *lepka* and *łopian* (VII); where *lepka* grows there is also *bobik* (VII); it can grow in the *Alnus* forest, *olsyna*, and in front of the forest in *biele* (white meadows) (III)	It grows in the zones further from the river (III, IV); in *smugi* (pasture), in places far from the river (II); in the middle place between river and forest; by mineral islands (VII)	Has three rounded leaves that are thick (all) but soft (VII); it used to grow in large aggregations in meadows (VII); it is very bitter (II)	It is really easy to mow, very light; where the *bobik* grows, the swath is extremely thick (VII)	Cattle and sheep eat it happily; when sheep finds *bobik* in a hay they are delighted; it is a highly valuable fodder (VII); cattle eat it (I); I did not give it to cattle because it was too bitter (IV); the mixture of *bobik, lepka* and *łopian*, which grow by the mineral islands, in hay has an amazing scent and sheep eat it directly in the air (very quickly); the hay with this mixture was deliberately rationed to sheep (VII)	Formerly, it used to be abundant (V, VII); back then, there were extensive fragments of *bobik* in meadows; today there is much less of it (VII); formerly, there was plenty of it, now there is much less because it is drier (I, IV)	It is a medicinal plant that is collected and sold; it used to be collected and sold fresh and wet (I, II, III, IV, V); it pays off to sell it; people used to harvest it directly from boats (IV); we use it at home to heal stomach pains (II)
	*kacak* (III) *kaczeniec* (II, III, IV, VI, VII) *łopian* (VII) *nikwiat* (II)	Marsh marigold-like Marsh marigold Burdock No-flower	*Caltha palustris* L	Grows in spring (II, III, VII); in the whole *biele* (all wet meadows) (III, VII); it grows when water is still there; after a long winter with snow, in spring there is plenty of them in the water (VII)	All *biele* (wet meadows) (II, III, VII)	It is quite fragile (III); it blossoms yellow (II,VII)		The leaves used to be harvested for pigs (VI); when cattle used to graze it in spring, they had a fatty, yellowish milk later on, it looked dyed; the milk trader always said that it is dyed with carrot (II, VII)	It needs high water in spring to grow; when the winter is long and snowy then there is a lot of water in the spring and *kaczeniec* is there (VII)	
	*osty* (II, III) *oset jeziorny* (IV)	Thistles Lake thistle	*Stratiotes aloides* L	Usually in the lakes (II, III, IV, VII); plenty of lakes have a name derived from *osty*		It breaks high through the surface (III); it blossoms white or blue (II); it blossoms white (II); it is so spiny that one can split one's legs during fishing; it is difficult to fish with a net because of it; even with a boat it is difficult to go through it (IV)	People used to mow it with a scythe to clean the lakes (IV)			
	*grążel* (II) *ryjki* (IV)	Water-lily Little snouts	*Nuphar lutea* (L.) Sm	On the whole surface of the lakes (IV)	We have a lot of it in lakes (IV)	It has roots like an arm (IV)				
	*grzebilja* (VII) *lilija* (VII)	Water-lily-like lily	*Nymphaea alba* L	Lakes (VII)	We have it in lakes (VII)	It blossoms white (VII)				
unaffiliated taxa	*lepka* (II, III, IV, VII)	Sticky one	*Galium uliginosum* L.*, Galium palustre* L	It grows between *rzeżucha* (III); it grows closer to the river, where there are floods from the river; it grows together with *bobik* in hollows by mineral islands (VII)	In the middle place between the river and forest, but closer to the river; by mineral islands (VII)	It blossoms white (III, VII); it creeps; it grows quite massively (VII); it winds over other plants and sticks to them (II, IV); it is a soft grass; it sticks to the fingers (IV, VII); when there are other plants around, it grows on them, if there are none, it creeps on the ground; it is somewhat heavy; it has little, fine leaves on the whole stem (IV); it is from *seradela* (bird's-foot) family; it blossoms for the first early mowing (VII)	It trails behind the scythe (VII)	It is a very good fodder grass (VII); cattle and sheep eat it happily (IV, VII)		
	*powójka* (III, IV, VII) *powojka* (IV)	Bindweed-like Bindweed-like	*Calystegia sepium* (L.) R. Br	It grows between tall grasses, like *bluszcz*, *rzeżucha*, reed (III, IV, VII); by the river it is very rare; a similar species grows in the crops fieds and climbs on the cereal (*Convolvulus arvensis*) (IV)	It does not grow by the river but in the zones further from the river (III, IV, VII)	It blossoms white (VII); it climbs high and winds over other tall plants (III, IV, VII); it is very difficult to break through this plant by hands (VII)		All animals like to graze it (III); cattle eat it like honey; it is a delicacy, a luxury for cattle (VII)	When in the 1980s we ceased mowing *biele* intensively, it started to grow everywhere (VII)	
	*kobylak* (II, III, IV, VI) *kobylak bielny* (II) *łopian* (VII) *szczaw koński* (VII)	*kobylak* wet meadow *kobylak* burdock giant water dock	*Rumex hydrolapathum* Huds	It grows in lowered places, in hollows (III) massively (VII); in muddy places; on the edges of the lakes (IV); a similar species grows in gardens and is called *szczaw* (dock) (II)	By the lakes and bodies of water (IV), everywhere in lowered wet meadows (III, VII)	It has very broad leaves, that is why sometimes they call it *łopian* (burdock) (III, VII); the leaves are more rounded; the whole plant is more green (VII); its roots are thick like an arm; its seeds are like groat (IV)		Cattle prefer not to eat it (IV, VII); it was mown for hay because cattle like it, too (III)	Formerly, there used to be plenty of it; when there was ice in winter, *kobylak* constantly moved with the pulled out ice to new places and would root there; now the years are dry, there is no ice that moves it to the new places, so it grows rarely	It is a medicinal plant and people collect and sell it
	*marchlak* (III) *marchwianka* (IV)	Carrot-like Carrot-like	*Oenanthe aquatica* (L.) Poir	It grows when a lot of water stays in spring (III); in 'river meadows' (III, IV); grows in muddy places (IV)	Grows in the 1st zone, on the other side of the river (III, IV)	It has a hollow inside the stem; one could stand on it like on a bridge (IV)		Cattle prefer not to eat it (IV)	In the years when the meadows stay dry it does not grow (III)	
	*truskawka* (VII)	Strawberry	*Fragaria vesca* L	It grows in *Alnus* forest, *olsyna* (VII)	*olsyna Alnus* forest (VII)	It blossoms white; grows very low, just over the ground (VII)		Pigs like to eat it; sheep with cattle used to graze it in autumn; sheep could only eat strawberries, does not need anything else; it is a delicacy for sheep (VII)	Back then, there used to be plenty of it in the forest (VII)	
	*rdest* (III, IV) *derdys* *derdes* (III)	Knotgrass Knotgrass-like Knotgrass-like	*Persicaria amphibia* (L.) Delarbre*, Persicaria hydropiper* (L.) Delarbre	It grows in wet meadows, in acidic places; similar species grow in crop fields (III)	Wet meadows and crop fields	It has willow-like leaves; there are a few types of *rdest*, some of them are smaller, some of them more massive (III); it is rare here (IV)		Cattle eat it only when they have to (IV)		
	*wilczy gnat* (III)	Wolf bone	*Sium latifolium* L	In slightly elevated places; small mineral islands in wet meadows (III)	In the 1st zone behind the river (III)	It has a very hard stem; it could blunt a freshly sharpened scythe; it blossoms white (III)		Cattle do not want to eat the old form (III)		
	*koluch* (IV)	Spine-like	*Sparganium erectum* L	It grows where *bobik* grows; by the ditches (IV); in *biele*, (wet meadows) (IV, VII); it grows in grass for the first mowing, not for the second (VII)	In the middle places between river and forest (VII); in further zones (IV)	It has flowers on one side (III); it is spiny (III, IV)			We did not have much of it back then, now we have more (VII)	
	*koczki* (III) *pałka* (IV)	*koczki* cattail	*Typha* spp.	In muddy wet meadows; on the edges of the lakes (IV)		It has a fluff that blows with the wind (IV)	People used to mow it with a scythe to clean the lakes (IV)			
	*skołojrza* (III, IV) *babka* (II)	*skołojrza* plantain	*Plantago media* L.*, Plantago lanceolata* L							Medicinal plants, leaves are collected and sold (II, IV); people used it *na obryw* (kind of folk disease) and put it on abscesses on the skin (IV)
	*świńska trawa* (III, IV)	Pig grass	*Polygonum aviculare* L							a medicinal plant, collected and sold
	*jaskrawiec* (IV)	*jaskier*—Polish botanical name of buttercup *jaskier-*like	*Ranunculus repens* L.*, Ranunculus flammula* L	Grows in *smugi* (pastures)		It is a burning plant—when put on skin on the inner part of the wrist, it makes a wound (IV)		Cattle do not eat it		
shrubs and trees	*krzewina* (all) *krzewa* *wici* *łozina* *rodzina łozinowatych* (VII)	Shrub-like Shrub-like Twine-like *łoza*—Polish common name for *Salix cinera* *łoza* family	Shrubby forms of *Salix* spp., e.g. *Salix cinerea* L	Wet meadows (VII); grows by the rivers and stabilises the edges (II, III)	In all wet meadows (VII); by the river and lakes (II, III)	It is a small *wierzba* (a tree from of *Salix*) (VI, VII); has broader leaves and darker bark (compared to *wierzba*) (III, V, VII); leaves are shiny (VII); there are a few types of *krzewina:* green, red, hard and soft; it is twisted (II)	It was used to build a structure under the haystack (III, IV, V, VI, VII); it was used for small poles stuck in the ground (platform) under the haystack; the branches were used as material layered on pole platforms (III, IV, V, VI, VII); straight trunks of harder *krzewina* were used as ballasting poles interposed on the top of the haystack (II, IV, VI, VII); branches used to mark the borders of the plots during haymaking in wetlands (IV, V); if you encounter it in the grass during mowing, then you immediately blunt your scythe, it is so hard (VII)		It started to grow everywhere when we stopped mowing meadows (VI, VII)	
	*krzewina* (all)	Shrub-like	Shrubby forms of *Alnus glutinosa* (L.) Gaertn.*, Frangula alnus* Mill				It was used to build a platform under the haystack (III, IV, V, VI, VII); it was used for small poles stuck in the ground under the haystack; used as material layered on the pole platform (III, IV, V, VI, VII); trunks used as ballasting poles on the top of haystack (IV, VI, VII); to mark the borders of the plots during mowing in wetlands (IV, V)			
	*wiklina* *złotooki* (IV)	Osier Golden eyes	Flexible types of *Salix* spp.			It is very flexible; there are three specific taxa of flexible *wiklina*: yellow, red (named *rózga,* rod) and green (IV)				
	*wierzba* (III, VII) *wierzbina* (V, VII)	Willow Willow-like	Tree form of *Salix* spp. e.g. *Salix alba* L	It does not grow on the edges of rivers and lakes as opposed to *krzewina* (VII)		There are a few species of it; has narrow leaves and fair bark (III); it grows very tall; is usually fragile (VII)				
	*brzoza* (III, IV, V, VI, VII) *brzezina* (I, II, VII) *brzózna* (II)	Birch Birch-like Birch-like	*Betula pendula* Roth.*, Betula pubescens* Ehrh	Grows in wet meadows and on sandy mineral islands (II)			Used for small poles stuck in the ground (platform) under the haystack (IV, VI, VII); trunks used as ballasting poles interposed on the top of the haystack (I, II, III, V, VI, VII); material used for *nosidła*—wooden rods used to carry hay to the haystack (III, IV, V); material for the scythe (V)	Cattle can eat the leaves		A medicinal plant; leaves are collected and sold (II, III, IV, V, VII)
	*kruszewina* (III, IV, VII) *wilczywe* *kruszyna* (VII)	Alder buckthorn-like Wolf-like Alder buckthorn	*Frangula alnus*	Grows in *olsyna Alnus*forest (III, IV); grows in forests (VII)	We have a lot of it in the *olsyna Alnus* forest (III)					A medicinal plant; we cut the branches, then peeled off the bark, then dried it out and sold it; wood used as firewood (VII)
	*dębina* (II, III, VII) *dębiak* (VII)	Oak-like Oak-like	*Quercus* spp.	In elevated places in wetlands such as mineral islands (II, III, VII)	On many mineral islands (VII)		Used for small poles under the haystack; branches put on the pole platform structure under the haystack; trunks used as ballasting poles on the tops of the haystacks (VII)		On some mineral islands it used to grow very well, but it wilted (VII)	
	*jegla* (III, VI, VII) *świerk* (all) *jegielka* (II, III) *choja* (IV)	*jodła—*Polish botanical name of *Abies alba*, so might be translated as *fir-like* spruce *fir-like* maybe from *choinka—*Christmas tree	*Picea abies* (L.) H.Karst	In sandy places (VII)		There are two specific taxa: red and white; red has a reddish wood after cut and darker needles, the white one has light wood and lighter needles (III, V, VII); it turns red when infected by woodworms; wood is more yellowish (VII)	The trunks were used for *nosidła* (wooden rods used to carry hay to the haystack) due to certain qualities of wood, which is strong, tough and light (all); used for small poles (platform) under the haystack or as ballasting poles interposed on the top of the haystack (IV); material used to make a scythe (V); the white one is better for *nosidła* because it is lighter when dries out (VII)			The roots were used to make *brodnie* (type of drag net for fishing) (VI)
	*olsza* (III, IV) *olcha* (II, IV, V, VII) *olszyna* (all) *olska* (II)	Alder Alder Alder-like Alder-like	*Alnus glutinosa*	In lowered places; muddy soils; in wet places it can form a forest (all); it grows everywhere in wet meadows (III)	Forms a forest in further zones (III, VII)		Used for small poles stuck in the ground (platform) under the haystack (I, III, IV, V); the branches were used as material layered on the platform (I, IV, V); trunks used as ballasting poles interposed on the top of the haystack (I, IV, V, VII); used for *nosidła,* wooden rods used to carry hay to the haystack (III, IV); it was not suitable to be used as ballasting poles put on the haystack because it was too fragile (VI)			
	*sośnina* (III) *sosna* (III, VI, VII)	Pine-like Pine	*Pinus sylvestris* L	In sandy places (VII)			Used for small poles stuck in the ground (platform) under the haystack (VII); used for *nosidła,* wooden rods used to carry hay to the haystack (III)			The roots were used to make *brodnie* (type of drag net for fishing) (VI)
	*lipa* (III, V, VI, VII)	Lime	*Tilia* spp.		We do not have a lot of it (III)		Branches put on the pole platform under the haystack in the winter time (VII); best material to use for *nosidła;* wooden rods used to carry hay to the haystack because it is strong and light wood (III)			When a cow ate too much of the young sprouts in the springtime, then it had inflammated urine with blood in it; it could be healed with 0,5 l of spirit vinegar; because of this, back then everyone had to have vinegar at home (VI)
	*leszczyna* (III, VII) *lescyna* (VIII)	Hazel Hazel-like	*Corylus avellana* L				Branches covered the pole platform under the haystack (VII)			
	*osa* (III, VII) *osika* (III)	Aspen-like Aspen	*Populus tremula* L	Also grows in wet meadows (III)		The wood is soft (VII)	The trunks used for *nosidła,* wooden rods used to carry hay to the haystack (III)			
	*grabina* (III, VII)	Hornbeam-like	*Carpinus betulus* L	On mineral islands (VII)	On many mineral islands (VII)					In grabina forest one can collect *Armillaria* mushrooms (III)
	*porzeczka czarna* (all)	Blackcurrant	*Ribes nigrum* L	Grows in olsyna *Alnus* forest (II, III)						A medicinal plants, leaves are collected and sold

The Roman numerals in parentheses indicate the village: I—Sośnia, II—Pluty, III—Brzostowo, IV—Rutkowskie, V—Kołodzieje, VI—Gugny, VII—Olszowa Droga

### Local plant knowledge

The local community distinguished more than 50 folk generic taxa (ethnospecies) among the plants growing in wetlands (Table 5). Some of the plants are named by and aggregated into groups that apply to a higher systematic domain such as a life form. As a rule, people differentiated groups of ‘grasses’, ‘shrubs’ and ‘trees’. Part of wetland species, which, according to people, do not have any farming value, were categorised as ***zioła*** ‘herbs’, ***rodzina ziół*** ‘herb family’ by a few knowledgeable informants. These plants, usually with colourfully blossoming flowers, frequently did not have generic names besides those used for medicinal purposes. This group of plants seems analogous to ‘grerb’—small herbaceous species, a life form distinct from grasses. Species with broad leaves growing on the water were sometimes classified into a group of ***kacaki***: *formerly, people named them kacaki* (III). Knowledge about these species was usually related to fishing, with the exception of *Menyanthes trifoliata*, which was a salient ingredient of hay. People also identified other generic taxa, which are not affiliated into any higher category.’Grasses’ (which also comprise some other grassland monocots such as sedges and rushes) are the utmost salient species in farming, therefore, there was the most extensive knowledge related to them. They are the prevalent ingredient of hay, dominate the landscape, and form the majority of the biomass collected from wetlands, so even if some of these ethnospecies did not have high fodder value, knowledge about them developed nevertheless. Similarly, ‘shrubs’ and ‘trees’ occurring in the area were salient species used as a source of timber and material for various necessary tools and constructions in traditional farming practices.

### Folk plant classification

A single folk generic taxon may refer to multiple botanical species of a similar habitat (***sitorz**** Juncus* spp., *Eleocharis palustris)*, similar morphological features (***hoszczka**** Equisteum fluviatile* and *Equisetum palustre*) and/or similar hay value (***rzeżucha**** Carex elata, Carex acutiformis* etc.). Sometimes one ethnospecies may comprise a few scientific species; however, it was not the whole plant individual that was identified as a folk taxon, but only the parts without flowering stems in the case of ***mózga**** Alopecurus geniculatus, Agrostis stolonifera* etc., or only the blossoming parts of the plant in the case of ***miotła**** Poa palustris* etc. The taxa of the ‘grasses’ category were predominantly distinguished by comparing their different morphological features (width of the leaves, height, or shape of the stem). In this way, the subgroup of ***trawy mieczowate*** ‘sword-like grasses’ (***bluszcz**** Glyceria maxima, ****tatarak**** Acorus calamus, ****kosak**** Iris pseudacorus*) was distinguished by a feature of the relatively broad leaves, which are much broader than those of other wetland ‘grasses’. People stated that some of the wetland taxa have comparable, twin plants occurring in a habitat other than wetlands, usually arable fields. These species were phrased by secondary lexemes (e.g. ***hoszczka polna**** Equisetum arvense*, ***miotła polna**** Apera spica-venti*), therefore, they might be classified as belonging to a lower domain—specific taxa—of the ethnospecies. Sometimes a single botanical species had clear regional semantic variations. *Phalaris arundinacea* was called ***jęczmianka*** by inhabitants from villages on the left bank of the Biebrza River, whereas inhabitants of the majority of the villages on the right riverbank and southern part of the LBB claimed that they have never heard this name and call this plant ***jemioła/niemioła***. Generally, characterisations of the species derives from traditional haymaking experience with the plants, e.g. what it was like to mow the plant with a scythe, to dry it, to rake it, to make a haystack out of it; how much hay the plant provides; how the plant was used during haymaking, etc. (Table 5).

### Distribution of species knowledge

When asked to freely list plant species growing in wetlands, people the most often cited ***rzeżucha*** (*Carex* spp.)—88.6% of informants, then ***mózga**** Agrostis stolonifera*, *Glyceria fluitans* etc.—77.1%, *Glyceria maxima*—74.3%, *Phalaris arundinacea*—68.6%, ***trzcina**** Phragmites australis*—65.7%, ***hoszczka*** wetland *Equisetum* spp.—65.7%, *Acorus calamus*—57.1%, ***bobrek**** Menyanthes trifoliata*—54.3%. A quarter of the informants mentioned ***okrąglica**** Carex appropinquata* etc. and ***kobylak**** Rumex hydrolapathum*. Slightly fewer informants listed ***mietlica*** blossoming *Poa palustris* etc. and ***kaczeniec**** Caltha palustris*, and one fifth of the informants listed: ***lepka**** Galium uliginosum, G. palustre*, ***osty**** Stratiotes aloides*, ***mięta**** Mentha aquatica*, ***powójka**** Calystegia sepium* and ***drabinka**** Potentilla anserina*. Other ethnospecies were mentioned less often. People did not usually mention shrubs and tree species in this part of the interview, as these plants were not included in hay.

### Species in habitats

Even if the landscape of the wetlands in the LBB seems to be continuously flat, local people in the first place described the habitat of the species by differentiating lower and higher places. For some plants, like *Glyceria maxima*, ***dołek*** ‘lowered place’ is applied to, the habitat that is lowered in relation to the level of the river. However, more often ***dołek*** related to hollows in wetlands—places where water used to stay longer, which are often differentiated from higher mineral islands and high structures formed by the plants, like tussocks. According to informants, in the hollows in wetlands there grow: *Phragmites australis*, ***mózga**** A. stolonifera,* etc., ***hoszczka*** wetland *Equisetum* spp., *Glyceria maxima, Acorus calamus, Menyanthes trifoliata*, ***lepka*** wetland *Galium* spp.*, Rumex hydrolapathum, ****olszyna**** Alnus glutinosa*. ***Rzeżucha**** Carex* spp. generally grows in higher places (forms tussocks), but also in lowered placed in front of mineral islands. Only in elevated places *Phalaris arundinacea, ****okrąglica**** Carex appropinquata, C. diandra*, ***mietlica*** blossoming steams of *Poa palustris, Deschampsia flexuosa*, ***tabuła**** Filipendula ulmaria, ****sitorz**** Eleocharis palustris, Juncus spp.*, ***wilczy gnat**** Sium latifolium*, ***dębina**** Quercus robur*, and ***grabina**** Carpinus betulus* occur*.* Some of the habitats were defined by the occurrence of accompanying species. For instance, a few informants said that: *Calystegia sepium* grows between ‘tall grasses’ like *Phragmites australis*, *Glyceria maxima, ****rzeżucha**** Carex* spp.; *Menyanthes trifoliata* can grow in hollows formed in front of mineral islands together with ***lepka**** Galium* spp. and *Rumex hydrolapathum,* providing a specific sort of very high quality hay good for sheep; *Glyceria maxima* can grow together with ***mózga*** or with *Phalaris arundinacea*. Species like ***mózga*** and *Phalaris arundinacea* dominate in hay from the second mowing. Conversely, ***koluch**** Sparganium erecum* prevails in hay from the first mowing.

### Species in the landscape

The location of certain plants within the wetland landscape is embedded in the local partitioning of the landscape (Fig. 4). As informants said, nearby the river and along waterbodies there grow *Acorus calamus, Iris pseudacorus, Phragmites australis* and *Typha* spp. The ‘river grasses’ of the 1st and 2nd zoning are generally dominated by ***mózga**** Agrostis stolonifera* etc.*, ****wilczy gnat**** Sium latifolium, ****marchlaki**** Oenanthe aquatica, Potentilla anserina* and ***hoszczka*** wetland *Equisetum* spp. A bit further away from the river, the second zone of the 1st zoning and the first zone in 2nd zoning are mainly constituted by *Glyceria maxima* and *Phalaris arundinacea.* In zones further from the river (1st zoning) or in between the 1st and 2nd zone (2nd zoning), where the ‘white meadows’ are, ***rzeżucha**** Carex* spp. prevails, accompanied by *Menyanthes, ****lepka*** wetland *Galium* spp.*, Calystegia sepium*, ***gęsie łapki**** Comarum palustre, Sparganium erectum*. The second zone (in 2nd zoning, VII) is dominated by ***siwucha**** Carex nigra*, *C. flava* etc. and ***tymotka*** flowering *Carex nigra,* occurring directly in front of the *Alnus* forest. Similarly, in the furthest zone from the river, by *Alnus* forests in the 3rd zoning (VI), ***okrąglica**** Carex appropinquata, C. diandra* prevails on the ‘peat meadows’. Many species can occur in *Alnus* forests, and include ***kruszewina**** Frangula alnus*, ***czarna porzeczka**** Ribes nigrum*, ***truskawka**** Fragaria vesca*, *Caltha palustris* and others.

### Species in management

Some species indicate a type and quality of vegetation that determines an area’s management. Vegetation constituted mainly by ***rzeżucha**** Carex* spp*.* was traditionally mown early, once a year, whereas ‘river meadows’ dominated by *Phalaris arundinacea* were traditionally mown twice a year. According to the informants, all species called *sitnik, sitarz* such as* Juncus* spp.*, Eleocharis palustris* etc. indicate acidic soil; when mown, they provide very bad quality hay. ***Okrąglica**** Carex appropinquata* etc. and ***siwucha**** Carex nigra* etc. indicate vegetation that is not dense enough to be mown using the common method ***na pokos***—from one side—and therefore should be mown ***na zbijaka***—from two sides—to provide a satisfyingly thick swath.

### Value of species used for hay

People expressed the high fodder value of a plant through its positive association with good quality and tasty food. The most valuable plants were most often described as ***słodkie trawy*** ‘sweet grasses’, less often ***tłuste trawy*** ‘fatty grasses’, or ***mające dużo białka*** ‘being rich in protein’. Conversely, plants that give low quality fodder were defined by all informants as ***kwaśne trawy***’sour grasses’. A few informants named them ***końskie trawy*** ‘horse-like grasses’, indicating that horses, believed to have the highest tolerance for different types of fodder, are more happy to eat those plants. Assumptions about the fodder value of the plants were based on observing if livestock eats the plant or not, how quickly it eats the plant, and what preferences the livestock has (for which species it reaches in the first place). Based on those observations, people also assessed the quality of the plants by comparing them to each other. When analysed qualitatively, the order of folk taxa ranked according to the value attributed to the given fodder by local people (based on observed cattle preferences) would be as follows. ***Mózga**** Agrostis stolonifera* etc.*, Menyanthes trifoliata, ****lepka*** wetland *Galium* spp. and *Calystegia sepium* were included in the best fodder unanimously by all informants. *Caltha palustris* in the spring and *Fragaria vesca* in the autumn provide very good seasonal fodder. The next best in terms of value would be *Glyceria maxima*, ***mietlica*** blossoming *Poa palustre* etc. and *Potentilla anserina*, which are eaten by cattle in any form. Meanwhile, *Phragmites australis* is a highly valuable plant only when it is young, and cattle does not eat it in the old stage. Similarly, *Phalaris arundinacea* could be ranked as a fodder plant of moderate value, since cattle eat only the very young plant or its leaves, avoiding the hard stem. However, it is good fodder for horses. Some but not all informants evaluated ***siwucha**** Carex nigra* etc., ***tymotka*** blossoming *Carex nigra*, ***hoszczka*** wetland *Equisetum* spp., *Rumex hydrolapathum* and *Sium latifolium* as good fodder*.* Next in rank could be ***rzeżucha***, a voluminous ethnospecies covering many *Carex* species. It was usually classified as low-quality fodder which, when young, can be alternatively eaten by cattle. The next could be ***rdest*** wetland *Persicaria* spp. and ***okrąglica**** C. appropinquata* etc.—plants eaten by cattle only when the animals have no choice of other fodder. Finally, there would be the group of plants which, according to people, are seldom eaten, like *Acorus calamus*, *Iris pseudacorus*, *Oenanthe aquatica*, ***sitorz**** Juncus* spp. etc., ***bociany**** Lysimachia vulgaris* etc., *Comarum palustre*, and ***tabuła**** Filipendula ulmaria*. The favourite plants for other livestock were as follows: ***mózga**** A. stolonifera* etc. is a delicacy for many animals, including cattle (especially calves), horses, pigs, sheep, and rabbits; *Menyanthes trifoliata*, ***lepka*** wetland *Galium* spp., *Rumex hydrolapathum* and *Fragia vesca* are the favourite fodder of sheep, whereas *Fragaria vesca* and the leaves of *Caltha palustris* are a delicacy for pigs.

### Increaser and decreaser species

The older informants shared the changes they observed in plant occurrence during their lifetimes. People noticed that in recent years a drought led to a decrease in the abundance of ***mózga**** A. stolonifera* etc., ***hoszczka*** wetland *Equisetum* spp. and *Menyanthes trifoliata*. People also observed that the occurrence of *Caltha palustris*, *Oenanthe aquatica* and *Rumex hydrolapathum* is depending on the temperature and snow cover in winter (directly related to amount of water in spring)—the colder and snowier the winter, the more abundant they are. According to many informants, in recent years, *Phragmites australis* started to prevail in vegetation. Firstly, they argued that, it used to be less common in the times when more cattle grazed in wetlands and trampled it. Secondly, it replaced ***rzeżucha**** Carex* spp., which does not tolerate mowing with machines, because they destroy its tussock structure. Discontinuation of mowing led to the increase of ***krzewina*** shrubby *Salix* spp. and *Calystegia sepium*. The increased abundance of *Phalaris arundinacea* was noticed in two villages; in one of these, the plant was observed to have replaced *Glyceria maxima.* In the two other villages where regular mowing by the river had been abandoned, more abundant *Glyceria maxima* was observed, together with an increase in the abundance of *Sparganium erectum* in one of the villages. Two informants spotted a local decrease of the ***okrąglica**** Carex appropinguata* etc. population, which seems to have been replaced by *Deschampsia flexuosa*.

### Shrubs and trees

Shrubs and trees used to serve as a source of timber for wintertime. They were also used to make e.g. platforms under haystacks, ballasting poles to cover haystacks, rakes, scythes, rods to carry hay, etc. As a rule, the type of the shrub or tree used, especially when it came to building a platform under a haystack, depended simply on its availability in the landscape. However, 77% informants indicated thin trunks of *Picea abies* as the best source of wood for rods used to carry ***kopy*** ‘small haystacks’ to a haystack. Spruce wood was described as lightweight and resistant, therefore perfect to carry heavy’small haystacks’. Alternatively, birch or alder wood could be used. As many as 71% of all informants indicated a freshly cut small birch tree as suitable to be used as a ballasting pole on a haystack due to its heaviness and elasticity, which made it possible to tie small branches together on the top of a stack. As many as 50% of informants also indicated alder as suitable for this purpose, however two people mentioned that it was a bit fragile. As many as 36% of informants listed ***krzewina*** shrubby forms of *Salix* spp., *Frangula alnus* etc.; however, they reasoned it was used only due to its common availability. Mostly hard species of ***krzewina*** shrubs, mainly *Salix* spp., then alder, birch, oak and pine were recommended for the small pales in haystack platforms. Branches of ***krzewina*** shrubs, mainly *Salix* spp., were commonly used as padding material.

## Discussion

### Local traditional knowledge of wetlands

Even though traditional land use of wetlands in the Biebrza Valley has been gradually ceasing since 1960s, the interviewed members of local community, particularly male representatives of the older generation, shared ecological knowledge on plants, landscape, and traditional management that was complex in dimensions and rich in detail. Women, being only partly engaged in traditional haymaking (hay raking and transporting), had much less wetland plant knowledge. As studies of Fawzi [64] and Mustonen [3] indicate, women can also be relevant knowledge holders, as long as they are fully engaged in land and resource management. A comparison of our results on traditional farming practices with the findings of Kiryło [78] from the same area revealed already forgotten elements of knowledge (like herding with a dog, recognition of more habitats). This fact as well as the limited knowledge on plants and livestock grazing preferences amid currently studied younger farmers confirm that since extensive traditional practices are being discontinued, knowledge on nature and its management is no longer generated, which leads to its degradation and loss [64, 65].

We assume, based on plant knowledge shared by older farmers, containing mainly qualities of plants in the context of personal farming experience, that direct contact with plants during haymaking (hand mowing with a scythe, hand raking of hay, building haystacks) and pasturing various livestock in wetlands are crucial factors in knowledge development. The more distanced the farmers are (literally—by machines), the poorer their knowledge, as we can see from the example of young farmers. Similarly to cattle herders [102, 103], farmers in the Biebrza Valley characterised plant ethnospecies by habitat, palatability and the preferences of different type of livestock, however they also added haymaking observations. Interesting remarks on assessing the fodder value of plants by traditional pig keepers (***svinjars***) ‘through the mouths of pigs’ [104] have been affirmed in the Biebrza Valley, where farmers do the same but ‘through the mouths of cattle’, which were the predominant type of livestock grazing in this area. Such knowledge evolves when farmers extensively observe livestock grazing in wetlands or consuming hay.

The basic division of plants into two opposable categories, the ‘sweet’ (palatable, preferred) and the ‘sour’ (unpalatable), is an old distinction among Polish peasants [105, 106]. It seems that the quality of hay assigned to the plant depends on the available vegetation type. For instance, cattle with access only to wetland vegetation highly prefer species like *Glyceria fluitans, Agrostis stolonifera, Glyceria maxima* [our study, 30, 34], whereas those grazing in the saline steppe with various drier habitats that include palatable *Festuca* grass would graze such species as alternative fodder in drought time [102].

### Knowledge on landscape and vegetation

The local, zonal perception of the wetland landscape is comparable to the scientific division into five vegetation zones in the LBB. However, the local community’s perception is strongly associated with management practices and hay quality, whereas the scientific division is based on vegetation type that is collateral to the river and connected with the dynamics of the Biebrza River flooding and groundwater flow [95]. In addition, wetland landscape is seen in its vertical dimension, having elevated and lowered places, which are the main criteria of defining folk plant habitats. Although the landscape of the Lower Biebrza Basin is extremely flat when seen from a distance, in fact it includes slightly higher sandy mineral islands [74] and elevated areas on the river edges [95]. Both a horizontal zoning pattern as well as a mosaic of elevated and lowered places have practical meaning for farmers and differentiate management units (of various grazing and mowing regime). Similarly the traditionally managed lowland floodplain of the Sava River in Lonjsko Polje Nature Park in Croatia consists of micro-depressions and micro-mountains formed by water flow, creating different habitats and vegetation [26]. We identified a modest number of wetland landscape elements in the inhabitants’ narratives compared to numerous folk habitats recognised in mountainous areas [107]. This is most likely a result of the uniform landform [108], the relatively species-poor vegetation of floodplain and fen meadows in the Biebrza Valley [109] and the fact that part of the knowledge might be already eroded when comparing our results with the findings of Kiryło [78].

### Wetland management practices

The traditional management regime in the Biebrza Valley was first of all flexible and conditioned on water level, similarly to management of the floodplain of the Sava River [26]. The first day of mowing was more or less fixed by church holidays—a marker of important activities associated with plants all over Poland. For example, Assumption Day and Corpus Christi Octave are days when herbs are blessed. They remind people to collect particular species of medicinal plants [110, 111]. The traditional farming calendar additionally depended on the location of the village, available habitats, vegetation, and type of livestock. The spatio-temporal management had a mixed character (grazing and mowing), as in floodplains of the Sava River [26]. In times of traditional farming all wetlands (besides forest) in the Biebrza Valley were mown for hay, which enabled scythe mowing. Generally, scythe mowing, introduced in Eastern Europe in the eleventh century [50, 112], is considered a major type of land use, which led to the widespread development of open wetlands and highly biodiverse fen meadows in Europe [32, 45, 113]. Open wetlands and forests in the Biebrza Valley were also grazed, mainly by cattle and horses, locally by sheep in the dry autumn season and by fowl on soft meadows by the river. In the 1980s the area was still described as a vast pasture [78]. In contrast to Pannonian wetlands in Central Europe, which were grazed extensively all year round [34], these wetlands were not grazed in winter (because of snow cover) or in summer, when they were intended to produce hay (at that time *Alnus* forest became alternative pasture). It is worth mentioning that in terms of creating open wetlands, in Europe, grazing by cattle is a 5–7 thousand-year older land-use type than mowing [32]. Pig grazing in wetland forests, which was locally practiced in the Biebrza Valley, was ceased in ca. 1970s. This practice was not only historically frequent in Europe [33, 34, 114] but present to this day in wet oak forests in floodplains of the Sava River [26, 104, 115].

Haymaking was strictly performed on the owned wetland plots, in opposition to grazing, for which wetlands were used communally, as was traditional also in the wetlands of the neighbouring Narew River [116] and the Balkan Sava River [26]. Interestingly, some of the farming techniques common in Biebrza, like mowing ***na pokos***, the second mowing called ***otawa***, and ***klepanie kosy na babce***, sharpening a scythe with hammer on special iron tool, were common in old traditional practices in other Slavic countries and beyond [114]; likewise ***wypas kolejką*** ‘pasturing in queue’ [26, 116]. However, these techniques were usually given different names.

Our study shows that even the poor-quality hay from fen meadows dominated by e.g. *Carex appropinquata* were used as fodder if no other type of hay was available, in opposition to the statement that historically low quality hay from fen meadows was used only for bedding [32, 33, 117]. However, the farming use of worse quality vegetation in the Biebrza Valley was less complex (usually one mowing, grazing by cattle and horses) than the diversified management regime of highly valued floodplain river meadows (as a rule mown twice, grazed by all possible types of livestock).

### Land use changes and conservation challenges

The intensification of farming in the Biebrza Valley, i.e. the gradual ceasing of scythe use and grazing livestock, led predominantly to the abandonment and overgrowing of wetlands in the LBB. Such land use transitions are considered to be the main threats to the biodiversity of wetlands in Europe [24, 32, 112]. For instance, as in wetlands of the Carpathian Basin [34] and coastal wetlands in Estonia [25], the lack of trampling cattle in the Biebrza Valley resulted in an uncontrolled spread of reed. Since the Biebrza National Park was established, many scientific research and conservation plans have been proposed and undertaken to protect the unique biodiversity and hydrology of the area [71, 118–120]. However, its protection status limited or banned some of the local community’s activities. For example, the burning of wetlands is strictly forbidden due to the risk of peat catching fire [121], even though the burning of tussock structured wetlands was traditionally practiced in early spring [78] and controlled burning is recommended as favouring the population of the Aquatic Warbler (a bird species under conservation; [122]). The local community used to extensively hunt for ducks, hares and otters [78], which is currently forbidden and recognised as poaching. Some traditional practices such as removing vegetation in the oxbows are also not continued. However, further research is needed to explore the current management of wetlands in the Biebrza Valley—which is defined not only by the conservation plans of the national park but also by EU agri-environmental regulations—and to propose recommendations on how traditional management and knowledge could be integrated into it.

## Conclusions

The research revealed and documented local traditional ecological knowledge on wetlands’ plants, vegetation, landscape and the mixed management regime that is still present among the local community of the Biebrza Valley. It confirms essentially and unquestionably the dominantly cultural origin and character of highly valued ecosystems in the studied area, hence the Biebrza Valley needs to be treated as a cultural landscape in any management endeavours. For this reason, the components and complexity of traditional farming and ecological knowledge of local people should necessarily be taken into consideration as inspiration for conservation management plans in the area, and collaboration with the local community should be undertaken in such activities. The research might give an incentive for further studies in other villages of the Biebrza Valley and in other areas of high environmental value and cultural origin.

## Data Availability

Voucher specimens for species were deposited in the herbarium of Warsaw University (WA). The datasets used and analysed during the current study are available from the corresponding author upon reasonable request.

## Abbreviations

LBB: Lower Biebrza Basin
TEK: Traditional Ecological Knowledge
LTEK: Local Traditional Ecological Knowledge
Biebrza NP: Biebrza National Park

## Footnotes

1 The Plant List (2013). Version 1.1. Published on the Internet; http://www.theplantlist.org/ (accessed 12 February 2021).

2 Plots in the Biebrza Valley are long and narrow due to the still present legacy of traditional land fragmentation along the length of plots, so that the plot became narrower and narrower but remained constant in length. In former times the long and narrow plots perpendicular to the river were assigned equally to people, so that everyone got better quality meadows by the river and the worse ones further from the river.

3 Information obtained from the last two elderly inhabitants of the village; they might have an incorrect memory of how ***smugi*** were used.
